# Genome-wide identification and analysis of DNA methyltransferase and demethylase gene families in *Dendrobium officinale* reveal their potential functions in polysaccharide accumulation

**DOI:** 10.1186/s12870-020-02811-8

**Published:** 2021-01-06

**Authors:** Zhenming Yu, Guihua Zhang, Jaime A. Teixeira da Silva, Mingzhi Li, Conghui Zhao, Chunmei He, Can Si, Mingze Zhang, Jun Duan

**Affiliations:** 1 Key Laboratory of South China Agricultural Plant Molecular Analysis and Genetic Improvement, South China Botanical Garden, Chinese Academy of Sciences, Guangzhou, 510650 China; 2 Economic Botany, Core Botanical Gardens, Chinese Academy of Sciences, Guangzhou, 510650 China; 3 Independent researcher, P. O. Box 7, Miki-cho post office, Ikenobe 3011-2, Miki-cho, Kagawa-ken 761-0799 Japan; 4 Biodata Biotechnology Co. Ltd, Hefei, 230031 China

**Keywords:** *Dendrobium officinale*, DNA methyltransferase, Stress, Water-soluble polysaccharides

## Abstract

**Background:**

DNA methylation is a conserved and important epigenetic modification involved in the regulation of numerous biological processes, including plant development, secondary metabolism, and response to stresses. However, no information is available regarding the identification of cytosine-5 DNA methyltransferase (*C5-MTase*) and DNA demethylase (*dMTase*) genes in the orchid *Dendrobium officinale*.

**Results:**

In this study, we performed a genome-wide analysis of *DoC5-MTase* and *DodMTase* gene families in *D. officinale*. Integrated analysis of conserved motifs, gene structures and phylogenetic analysis showed that eight DoC5-MTases were divided into four subfamilies (DoCMT, DoDNMT, DoDRM, DoMET) while three DodMTases were divided into two subfamilies (DoDML3, DoROS1). Multiple *cis*-acting elements, especially stress-responsive and hormone-responsive ones, were found in the promoter region of *DoC5-MTase* and *DodMTase* genes. Furthermore, we investigated the expression profiles of *DoC5-MTase* and *DodMTase* in 10 different tissues, as well as their transcript abundance under abiotic stresses (cold and drought) and at the seedling stage, in protocorm-like bodies, shoots, and plantlets. Interestingly, most DoC5-MTases were downregulated whereas DodMTases were upregulated by cold stress. At the seedling stage, *DoC5-MTase* expression decreased as growth proceeded, but *DodMTase* expression increased.

**Conclusions:**

These results provide a basis for elucidating the role of DoC5-MTase and DodMTase in secondary metabolite production and responses to abiotic stresses in *D. officinale*.

**Supplementary Information:**

The online version contains supplementary material available at 10.1186/s12870-020-02811-8.

## Background

Cytosine DNA methylation is an evolutionarily conserved epigenetic modification that is critical for the regulation of gene expression, imprinting, plant development, fruit ripening, control of transposon activity, and stress responses [1–3]. Mammalian methylation mainly occurs in symmetric CG contexts, while plant DNA methylation occurs in all sequence contexts, including CG, CHG, and CHH, where H represents A, C, or T [4]. In plants, DNA methylation is divided into two categories: maintenance of DNA methylation and de novo DNA methylation. Plant cytosine DNA methylation can be maintained by METHYLTRANSFERASE1 (MET1), CHROMOMETHYLASE3 (CMT3), and DOMAINS REARRANGED METHYLASE2 (DRM2) and CMT2 at CG, CHG, and CHH sites, respectively [2–6]. In addition, DRM is primarily responsible for de novo DNA methylation in all sequence contexts (CG, CHG, and CHH) via the DNA methylation pathway directed by 24-nt small interfering RNAs [3, 4].

Generally, the precise status of DNA methylation in plants depends on the dynamics of antagonistic DNA methylation and demethylation. Compared to DNA methylation, whose catalyzation is determined by a single DNA methyltransferase such as MET1, CMT2, CMT3 or DRM2, active DNA demethylation requires a series of enzymes involved in excision of 5-methylcytosine in the CG, CHG and CHH contexts [2]. In plants, this process is initially catalyzed by a bifunctional DNA glycosylase, including REPRESSOR OF SILENCING1 (ROS1), DEMETER (DME), DEMETER-like 2 (DML2), or DEMETER-like 3 (DML3), through the base-excision repair pathway [1, 3].

Increasing evidence demonstrates that plant DNA methylation and demethylation are closely associated with various environmental stresses, including cold [7], drought [8], heat [9], heavy metal [10], salt [11], and ultraviolet stresses [12]. Additionally, DNA methylation and demethylation also play an indispensable role in regulating fruit development and ripening [3, 13, 14], as well as secondary metabolism [15–17]. Differences in anthocyanin levels between *Malus domestica* ‘Granny Smith’ and ‘Golden Delicious’ are attributed to differential DNA methylation levels of the v-myb avian myeloblastosis viral oncogene homolog (MdMYB1) promoter in both cultivars, altering the differential accumulation of MdMYB1-specific transcript levels, and in turn affecting the formation of red pigmentation in apple skin [18]. Similarly, temperature-dependent DNA demethylation is a key factor of postharvest temperature-affected anthocyanin biosynthesis in the flesh of an originally white-fleshed peach (*Prunus persica*). Increased expression levels of the anthocyanin biosynthesis-related genes, including *phenylalanine ammonia-lyase* (*PpPAL*), cinnamate 4-hydroxylase (*PpC4H*), *4-coumarate co-enzyme A ligase* (*Pp4CL*), *flavanone 3-hydroxylase* (*PpF3H*), *flavonoid 3′-hydroxylase* (*PpF3’H*), *dihydroflavonol 4-reductase* (*PpDFR*) and *anthocyanin synthase* (*PpANS*), as well as a transcription factor gene, *basic helix-loop-helix* 3 (*PpbHLH3*), were associated with lower methylation levels in the promoters of these genes [19]. Additionally, OsROS1-mediated DNA demethylation in the rice endosperm restricts the number of aleurone cell layers, and thus the OsROS1 mutant contains more non-starch polysaccharides, lipids, proteins, vitamins, and minerals than the wild type [20]. Given that DNA methylation and demethylation are essential for many biological processes in plants, *C5-MTase* and *dMTase* genes have already been identified and characterized in several plant species, such as thale cress (*Arabidopsis thaliana*) [21], peanut (*Arachis hypogaea*) [22], rapeseed (*Brassica napus*) [23], castor bean (*Ricinus communis*) [24], tomato (*Solanum lycopersicum*) [25], and tea plant (*Camellia sinensis*) [26]. However, to date, no research has yet focused on the identification and analysis of *C5-MTase* and *dMTase* genes in the orchid, *Dendrobium officinale* Kimura et Migo, based on genome-wide analyses.

*D. officinale* belongs to the Orchidaceae, and is a time-honored tonic food and traditional Chinese herbal medicine because of its abundant active secondary metabolites found in stems, especially water-soluble polysaccharides (WSPs) that exhibit anti-inflammatory, antitumor and antioxidant activities [27, 28]. Previous studies indicated that *D. officinale* WSPs can improve abiotic stress (e.g. drought and salt stress) tolerance, because they can act as compatible solutes and enhance water uptake from an osmotically stressed environment [29–31]. In addition, most epiphytic orchids adhere tightly to the surface of tree bark or rocks, so that they usually experience environmental stresses (e.g. cold and heat stress, and water deficit). Hence, *D. officinale* has evolved desirable qualities for mitigating harsh habitats, for instance Crassulacean acid metabolism, pseudobulbs, succulent storage organs, and thick leaves [32]. The *D. officinale* reference genome is publicly available [33, 34]. Several bioactive compounds with medicinal effects, such as polysaccharides and alkaloids, have been identified and functionally analyzed [28–30, 35, 36]. However, no information on *C5-MTase* and *dMTase* genes in *D. officinale* is available. In order to investigate whether DNA methylation and demethylation are involved in the regulation of the biosynthesis of active compounds, genome-guided discovery and characterization of *C5-MTase* and *dMTase* genes in *D. officinale* were performed. Our study provides valuable information for future functional characterization of these two epigenetic regulatory enzymes in plants of the orchid *D. officinale*, as well as for analyzing their evolutionary relationships within the entire plant kingdom.

## Results

### Genome-wide identification and structural analysis of *C5-MTase* and *dMTase* genes in *D. officinale*

Blast analysis of reported *A. thaliana* and rice C5-MTase and dMTase proteins against the whole *D. officinale* genome resulted in the identification of eight DoC5-MTase and three DodMTase proteins (Table 1). The eight *DoC5-MTase* genes (*DoMET1*, *DoCMT1*, *DoCMT2*, *DoCMT3*, *DoDRM1*, *DoDRM2*, *DoDRM3* and *DoDNMT2*) code proteins composed of 324 (DoDNMT2) to 1534 (DoMET1) amino acids, similar to *AtC5-MTases* (383 to 1534) [21]. The MW of DoC5-MTases varies from 36.43 to 173.71 kDa with a pI ranging from 5.00 to 9.16 (Table 1). The full-length of the three DodMTase proteins varies from 1380 (DoDML3) to 1903 (DoROS1b) amino acids with an open reading frame (ORF) ranging from 4143 to 5712 bp, relative MW between 156.56 and 212.56 kDa, and a pI ranging from 6.70 to 7.85 (Table 1). The highest GRAVY value was observed for DoDNMT2 (− 0.235) and the lowest value for DoCMT1 (− 0.626), indicating that all *D. officinale* DoC5-MTase and DodMTase proteins are hydrophilic (Table 1). According to their putative subcellular localization, DoDRM1 was located in the cytoplasm, whereas the other 10 proteins were located in the nucleus. These findings are consistent with results in *Ricinus communis* in which different DRM members are located in different organelles [24]. Apart from the nuclear localization signals, RcDRM3 may be located in the mitochondrion, and RcDRM1 may be located in the chloroplast or mitochondrion [24].

**Table 1:** Basic features of *C5-MTase* and *dMTase* genes identified in *D. officinale*

Gene name	Gene ID	Chromosome location	ORF^**a**^ (bp)	AA^**b**^ (aa)	MW^**c**^ (kDa)	GRAVY^**d**^ value	pI^**e**^	Intron^**f**^	Predicted subcellular localization
*DoMET1*	Dca001068	Scaff_scaffold_716	4605	1534	173.71	-0.414	5.76	18	Nuclear
*DoCMT1*	Dca005086	Scaff_scaffold_370	2883	960	107.69	-0.626	6.24	26	Nuclear
*DoCMT2*	Dca003680	Scaff_scaffold_390	975	324	36.43	-0.238	7.49	7	Nuclear
*DoCMT3*	Dca021108	Scaff_scaffold_883	2670	889	100.30	-0.543	5.34	28	Nuclear
*DoDRM1*	Dca019831	Scaff_scaffold_547	1425	474	53.70	-0.367	9.16	5	Cytoplasmic
*DoDRM2*	Dca002179	Scaff_scaffold_1220	1749	582	65.32	-0.469	5.00	12	Nuclear
*DoDRM3*	Dca020469	Scaff_scaffold_726	1473	527	59.32	-0.416	6.18	17	Nuclear
*DoDNMT2*	Dca009405	Scaff_scaffold_264	1053	350	40.11	-0.235	6.60	13	Nuclear
*DoDML3*	Dca014673	Scaff_scaffold_807	4143	1380	156.56	-0.597	7.85	39	Nuclear
*DoROS1a*	Dca024052	Scaff_scaffold_489	5568	1855	207.52	-0.592	7.01	37	Nuclear
*DoROS1b*	Dca020504	Scaff_scaffold_993	5712	1903	212.56	-0.603	6.70	34	Nuclear

^a^ORF, open reading frame; ^b^AA, amino acid; ^c^MW, molecular weight; ^d^GRAVY, grand average of hydrophobicity; ^e^pI, theoretical isoelectric point; ^f^Intron, number of introns. Subcellular localization was predicted by Plant-mPLoc (http://www.csbio.sjtu.edu.cn/bioinf/plant-multi/)

**Fig. 1:**
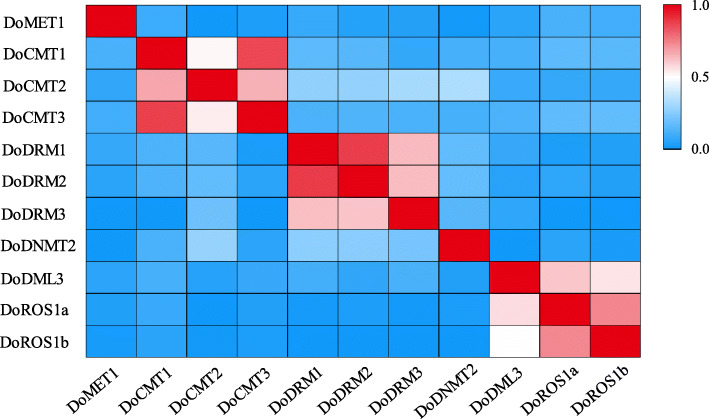
Sequence similarity of DoC5-MTases and DodMTases in *D. officinale*. Color scale on the right indicates the correlation of two proteins. Dodger blue represents a weak correlation, while deep red represents a strong correlation for these proteins

**Fig. 2:**
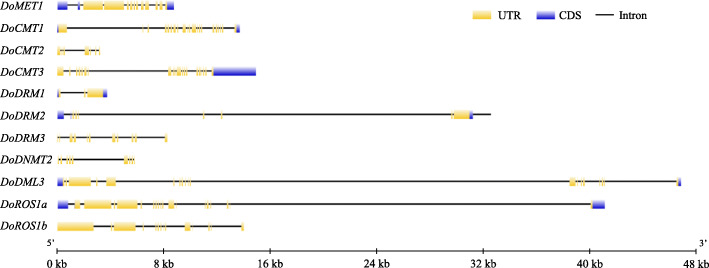
Gene structures of *DoC5-MTase* and *DodMTase* genes in *D. officinale*. Introns are presented by lines. UTR and CDS are indicated by filled yellow and blue boxes, respectively

The sequence similarity of *D. officinale* DoC5-MTase and DodMTase proteins was also analyzed. Compared with DodMTase proteins, DoC5-MTase proteins displayed low identity with each other, indicating that different DoC5-MTase proteins might possess diverse functions. DoCMT1 and DoCMT3, as well as DoDRM1 and DoDRM2, shared significantly higher similarities than other DoC5-MTase proteins. Among DodMTase proteins, sequence similarity between DoROS1a and DoROS1b was highest (Fig. 1). These results suggest that some sequences may be duplicated in *DoC5-MTase* and *DodMTase* genes. In addition, the coding region of *DoC5-MTase* genes was interrupted by 5–28 introns, while that of *DodMTase* was interrupted by 34–39 introns. Among these, the *DodMTase* gene with the largest number of introns is *DoDML3*, while *DoDRM1* contains the least introns (Fig. 2).

**Fig. 3:**
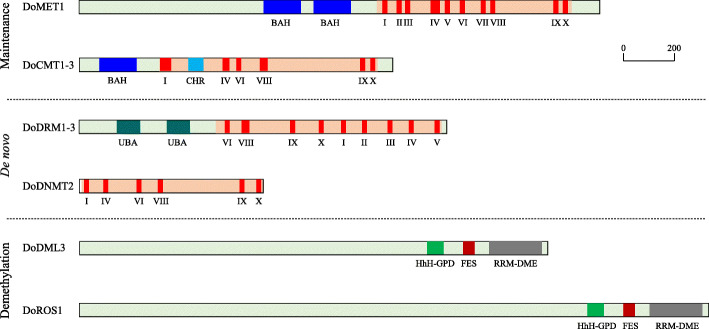
Schematic structures of DoC5-MTase and DodMTase proteins in *D. officinale*. BAH, bromo adjacent homology domain; CHR, chromo domain; FES, 4Fe-4S cluster domain; HhH-GDP, helix-hairpin-helix-Gly-Pro-Asp domain; RRM-DME, RNA-recognition motif demethylase PF15628 in Demeter; UBA, ubiquitin-associated domain PF00627. I to X, the conserved motifs in C5-MTase

Although the catalytic C-terminal domains with conserved domains were ubiquitous in all identified DoC5-MTase and DodMTase proteins, the N-terminals showed diverse combinations of conserved domains in each subfamily. Structural analysis revealed that eight DoC5-MTase proteins were divided into four groups on the basis of the diverse structural features comprising their primary structure: DoMET1 comprised the MET group that harbored two BAH domains in the N-terminal, DoCMT1, DoCMT2 and DoCMT3 comprised the CMT group that harbored only one BAH in the N-terminal, DoDRM1, DoDRM2 and DoDRM3 comprised the DRM group that harbored the UBA domain in the N-terminal, while DoDNMT2 was the sole member in the DNMT group without an N-terminal conserved domain. All of the DoC5-MTase proteins contained the carboxyl C-terminal catalytic domain with the conserved motifs I, IX, VI, VIII, IV and X aligned in a specific order, whereas the divergence of the N-terminal domains could be critical for distinct roles of different DoC5-MTase proteins (Fig. 3, Figure S1, S2, S3, S4, S5 and S6). In addition, DodMTase proteins contained three conserved domains, HhH-GPD, FES and RRM-DME. These results suggest that the majority of domains in DodMTase proteins are highly conserved.

### Sequence alignment and conserved motif analysis of C5-MTases and dMTases in *D. officinale*

**Fig. 4:**
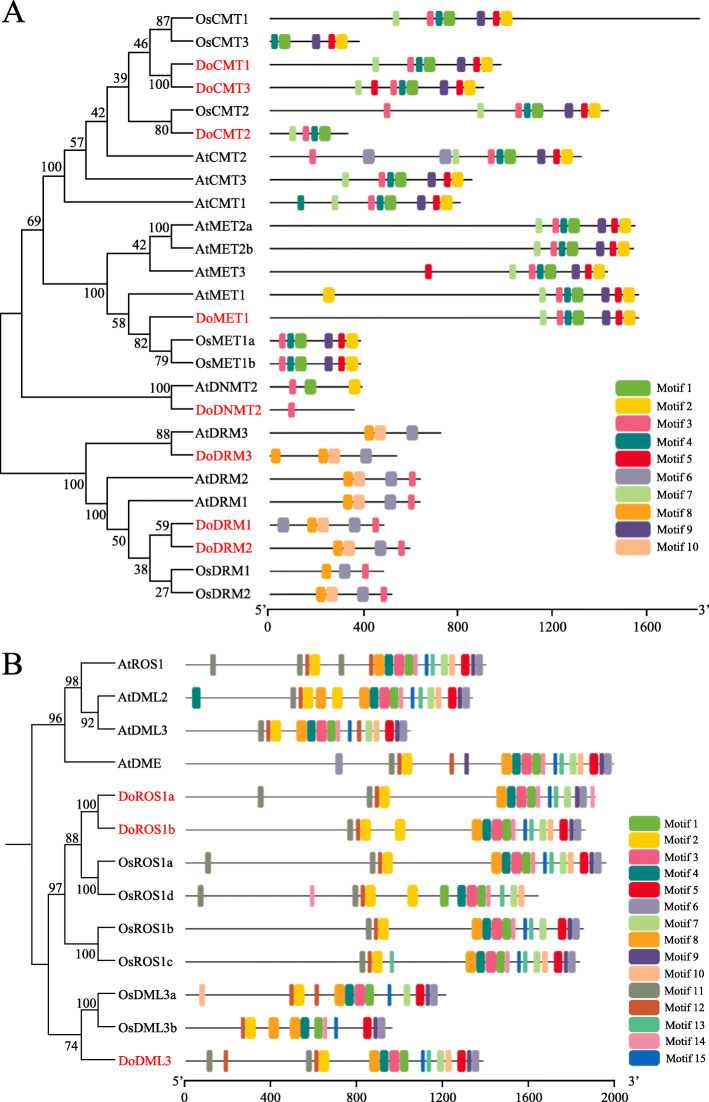
Conserved motifs of C5-MTase (**a**) and dMTase (**b**) proteins from *D. officinale*, *A. thaliana* and *O. sativa*

To further explore the conservation and divergence of DoC5-MTase and DodMTase proteins, the MEME motif search tool was used to identify conserved motifs in *D. officinale* and *A. thaliana*, a dicotyledonous model plant, and *O. sativa*, a monocotyledonous model plant. Among these C5-MTase proteins, a total of 10 conserved motifs were identified. The length of motifs ranged from 29 to 50 amino acids, and the number of motifs varied between 1 (DoDNMT2) and 10 (AtCMT2). Motifs 6, 8 and 10 were highly conserved in the DRM subfamily. Motifs 1, 2, 3, 4, 5 and 9 were located in the C-terminal region, and were the major conserved motifs in the CMT and MET subfamilies. Apart from OsCMT3, AtDRM3 and DoDRM3, motif 3 was located in all C5-MTase proteins (Fig. 4a, Figure S7, Table S2).

Accordingly, the distribution of dMTase protein motifs in *A. thaliana*, *D. officinale* and *O. sativa* were analyzed using the MEME suite, and 15 conserved motifs, designated as motifs 1 to 15, were identified. The length of motifs ranged from 18 to 50 amino acids and the number of motifs in each dMTase protein varied between 10 and 15. Motifs 1, 2, 4 and 12 were highly conserved in all dMTase proteins. Motifs 3, 6, 7, 8, 9 and 15 were found in 12 of 13 dMTase proteins, motifs 5, 11 and 14 were observed in 11 of 13 dMTase proteins, and motifs 10 and 13 were observed in 10 of 13 dMTase proteins (Fig. 4b, Figure S8, Table S3). It could be inferred that motifs common to both C5-MTase and dMTase proteins are probably associated with conserved biological functions, but those specific to a few proteins may be related to gene-specific functions.

### Phylogenetic analysis of C5-MTases and dMTases in *D. officinale* and other plant species

**Fig. 5:**
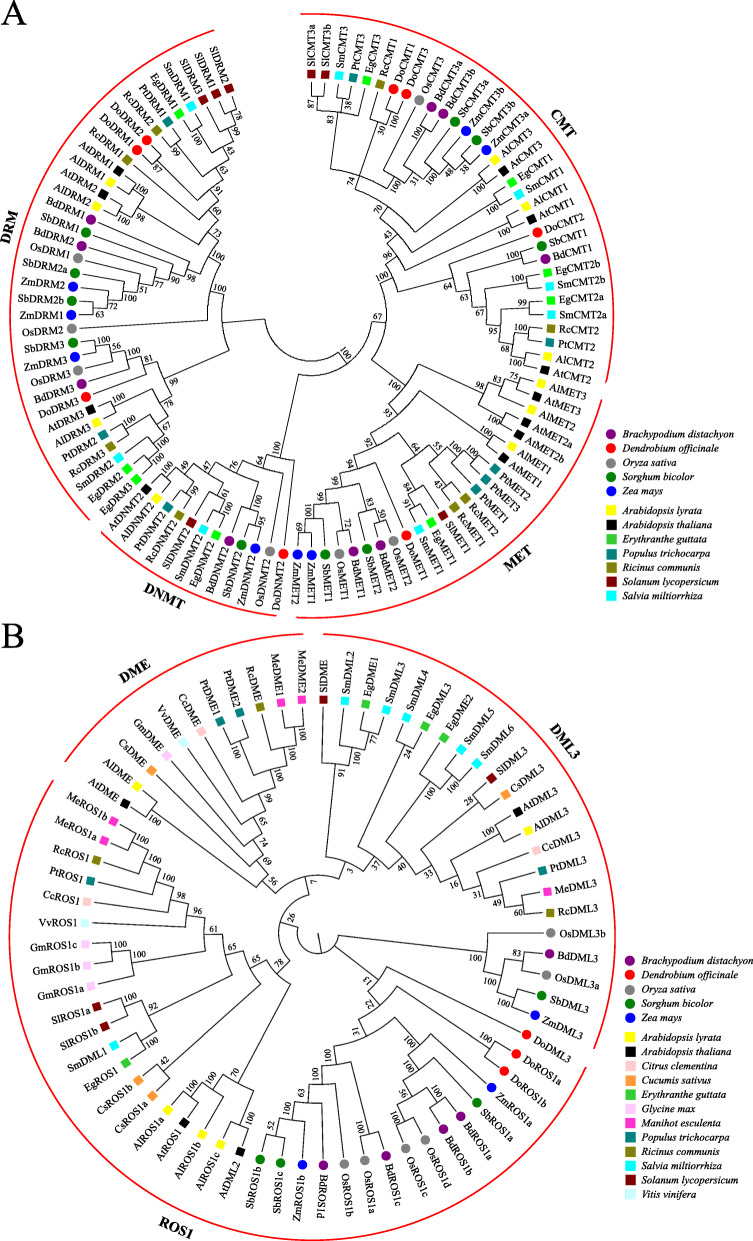
Phylogenetic analysis of the C5-MTase (**a**) and dMTase (**b**) proteins. Dicots and monocots are shown by a circle and square, respectively. Al, *Arabidopsis lyrata*; At, *Arabidopsis thaliana*; Bd, *Brachypodium distachyon*; Cc, *Citrus clementina*; Cs, *Cucumis sativus*; Do, *Dendrobium officinale*; Eg, *Erythranthe guttata*; Gm, *Glycine max*; Me, *Manihot esculenta*; Os, *Oryza sativa*; Pt, *Populus trichocarpa*; Rc, *Ricinus communis*; Sm, *Salvia miltiorrhiza*; Sl, *Solanum lycopersicum*; Sb, *Sorghum bicolor*; Vv, *Vitis vinifera*; Zm, *Zea mays*

To elucidate the phylogenetic relationship among C5-MTases in plants, 103 C5-MTase protein sequences from five monocotyledons (*B. distachyon*, *D. officinale*, *O. sativa*, *S. bicolor* and *Z. mays*) and seven dicotyledons (*A. lyrata*, *A. thaliana*, *E. guttata*, *P. trichocarpa*, *R. communis*, *S. lycopersicum* and *S. miltiorrhiza*) were used to construct corresponding phylogenetic trees (Table S4). C5-MTases were naturally grouped into four subfamilies, including 35, 32, 12 and 24 members in CMT, DRM, MET and DNMT groups, respectively (Fig. 5a). Compared to the DRM subfamily, the MET, CMT and DNMT subfamilies were more similar and belonged to the same cluster. The DRM subfamily was the largest in the phylogenetic tree, whereas the DNMT subfamily was the smallest and only contained 12 members from the 12 plant species. The MET subfamily harbored DoMET1 and could be further divided into dicot and monocot groups. Similarly, CMT, DRM and DNMT2 subfamilies could also be divided into a monocot group and a dicot group.

Accordingly, 69 dMTase proteins were clustered into three orthologous clades, designated as DME, DML3 and ROS1 groups, which harbored 11, 22 and 36 members, respectively (Fig. 5b, Table S5). According to the constructed phylogenetic tree, the ROS1 and DML3 groups could be further divided into the monocot and dicot subgroups. However, the DME group was only restricted to dicots, indicating that DME might be phylogenetically monophyletic in dicots. Overall, these findings suggest that C5-MTase and dMTase proteins might exercise different functions in monocots and dicots.

### Protein-protein interaction of DoC5-MTase and DodMTase

**Fig. 6:**
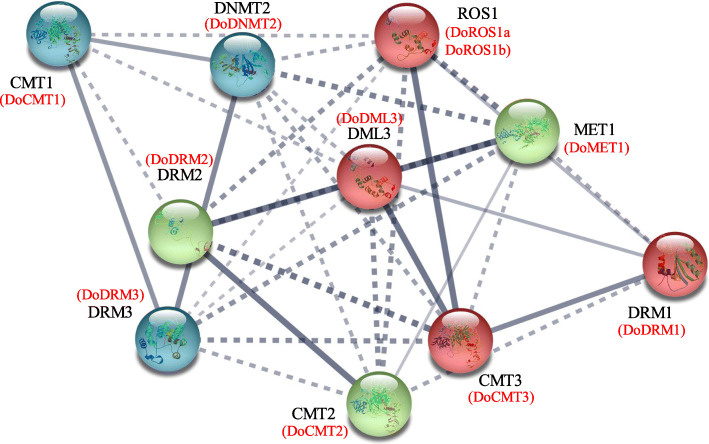
Potential protein-protein interaction network of DoC5-MTases and DodMTases. The protein interactions are weighted by a correlation coefficient. The dotted lines represent a relatively weak relationship while the solid lines indicate a relatively strong relationship, and the thicker lines indicate stronger correlations

Using *A. thaliana* homologues, a protein-protein interaction network of DoC5-MTase and DodMTase proteins was constructed with the STRING 11 tool. Consequently, DoC5-MTase and DodMTase were aligned to the corresponding *A. thaliana* orthologous proteins (Fig. 6). DoROS1a and DoROS1b were highly homologous to AtROS1, with 56 and 42% identity, respectively. A strong interaction among CMTs, DRMs and METs was observed at a given confidence level (0.70), indicating that they might regulate the overall DNA methylation levels via a protein-protein interaction network or by forming protein complexes. ROS1s and DMLs interact with C5-MTases, especially CMTs and DRMs, indicating that global DNA methylation levels might be dynamically accommodated by both C5-MTase and dMTase. Consequently, C5-MTase and dMTase might form a reciprocal negative feedback loop, which dynamically affects the overall methylation levels.

### Analysis of *cis*-acting elements in *DoC5-MTase* and *DodMTase* genes

**Fig. 7:**
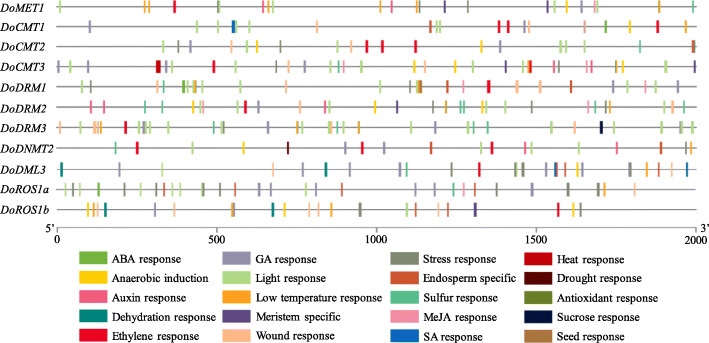
Prediction of *cis*-elements in the 2000-bp upstream regulatory regions of *DoC5-MTase* and *DodMTase* genes. Different *cis*-responsive elements are represented by different colored boxes

Plant growth and development are regulated by different *cis*-regulatory elements in genes. Here, various *cis*-acting elements, containing hormone-sensitive, light-responsive, stress-responsive, tissue-specific and other elements were mined and analyzed from the 2000-bp upstream regulatory regions of *DoC5-MTase* and *DodMTase* genes using the PlantCARE web site (Fig. 7, Figure S9, Table S6). Tissue-specific elements (12/244), including in the endosperm (6/12), shoot and root meristem (5/12) and seed (1/12), were found in the putative promoters of *DoC5-MTase* and *DodMTase* genes. Hormone-responsive elements (70/244), in response to ABA (18/70), auxin (9/70), ethylene (6/70), GA (18/70), MeJA (18/70) and SA (1/70), were also widely observed. Multiple abiotic and biotic stress-related elements (69/244), including in response to anaerobic induction (11/69), dehydration (6/69), drought (1/69), heat (2/69), low temperature (17/69), stress (15/69) and wounding (17/69), were largely enriched. These findings suggest that the *DoC5-MTase* and *DodMTase* genes might play an important role in response to cold and drought stresses in *D. officinale*.

### Transcript abundance analysis of *DoC5-MTase* and *DodMTase* genes in *D. officinale*

**Fig. 8:**
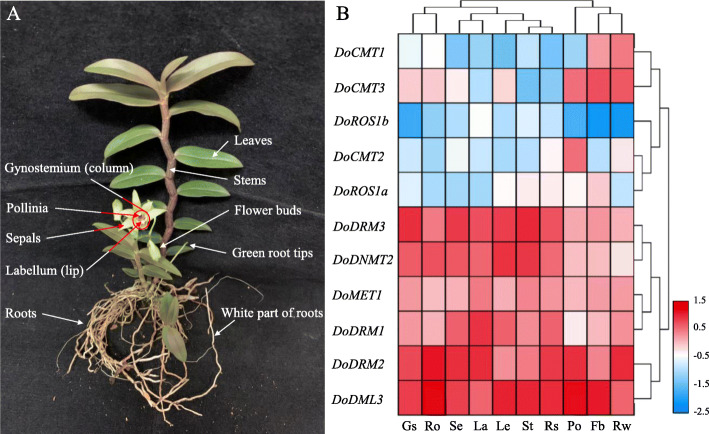
Tempo-spatial expression patterns of *DoC5-MTase* and *DodMTase* genes in different organs in *D. officinale*. **a** Different *D. officinale* organs were used in this study. **b** Transcript levels of *DoC5-MTase* and *DodMTase* genes in ten *D. officinale* organs. The log2-transformation of the average of expression values were used to generate the heat map with TBtools software. Blue and red in the color scale indicate low and high transcript expression, respectively. Fb, flower buds; Gs, gynostemium (column); La, labellum (lip); Le, leaves; Po, pollinia; Ro, roots; Rt, green root tips; Rw, white part of roots; Se, sepals; St, stems

As the main participants of DNA methylation, C5-MTases and dMTases play important roles in plant growth and development. To preliminarily investigate the biological function of DoC5-MTases and DodMTases, the transcript abundance of eight *DoC5-MTase* and three *DodMTase* genes in flower buds, green root tips, gynostemium (column), labellum (lip), leaves, pollinia, sepals, stems, roots and the white part of roots of two-year-old, field nursery-grown *D. officinale* plants was determined. Results indicate that all *DoC5-MTase* and *DodMTase* genes could be determined in the tested organs, but significant differential expression patterns were observed. According to hierarchical clustering (Fig. 8b), the transcript abundance of *DoC5-MTase* and *DodMTase* genes could be broadly split into two groups, the low- and high-expression groups. Members of the low-expression group (*DoCMT1*, *DoCMT2*, *DoCMT3*, *DoROS1a* and *DoROS1b*) generally maintained a relatively low level of expression in most tissues with a FPKM value ranging from 0.64 to 9.36. However, multiple genes were highly expressed in specific organs, such as *DoCMT1* in flower buds and the white part of roots, *DoCMT2* in pollinia, and *DoROS1a* in flower buds. Additionally, members of the high-expression group (*DoDRM1*, *DoDRM2*, *DoDRM3*, *DoMET1*, *DoDML3* and *DoDNMT2*) were expressed at high levels in all 10 organs with a FPKM value ranging from 12.55 to 47.47, especially *DoDRM2* and *DoDML3*, suggesting that these genes might play essential roles in plant growth and development. Furthermore, a total of 81.82% (9/11) of the *DoC5-MTase* and *DodMTase* genes showed intermediate and high expression levels in flower buds, pollinia and the white part of roots, compared with 63.64% (7/11) in the gynostemium and roots.

### Expression patterns of *DoC5-MTase* and *DodMTase* genes in response to environmental stresses

**Fig. 9:**
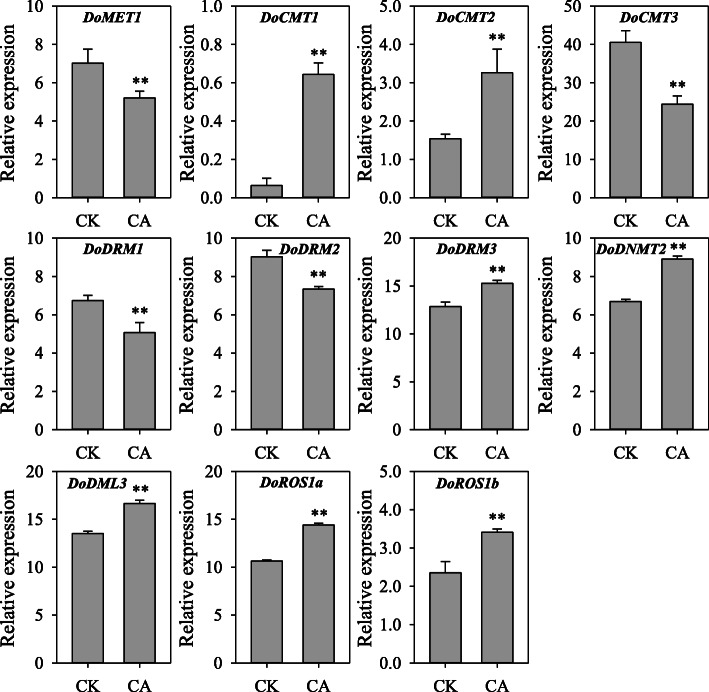
Transcript levels of *DoC5-MTase* and *DodMTase* genes in response to cold stress. Relative expression was quantified by previous *D. officinale* RNA-seq data [34] subjected to ambient (CK, 20 °C) and cold (CA, 0 °C) treatment for 20 h. Error bars indicate the mean ± SD (standard deviation) of three individual experiments, and were quantified with three independent replicates. Asterisks above bars indicate a significant difference between CK and CA at *p* < 0.01 based on the Student’s *t*-test. CA, cold treatment at 0 °C; CK, the control at 20 °C

*D. officinale* is an epiphytic orchid that takes root on trunks and cliffs, and is usually exposed to diverse environmental stresses, especially drought and cold stress. To investigate the responses of *DoC5-MTase* and *DodMTase* genes under cold stress, *D. officinale* RNA-seq data [37] subjected to ambient (CK, 20 °C) and cold (CA, 0 °C) treatment for 20 h was analyzed. Cold treatment differentially regulated the expression of *DoC5-MTase* and *DodMTase* genes (Fig. 9). Compared to the non-acclimated controls, transcript levels of *DoCMT3*, *DoDRM1*, *DoMET1* and *DoDRM2* were suppressed between 1.12- and 2.48-fold. In contrast, *DoDML3*, *DoDNMT2*, *DoROS1a*, *DoDRM3*, *DoCMT1*, *DoCMT2* and *DoROS1b* were enhanced between 1.14- and 7.55-fold, the highest for *DoCMT1* and the lowest for *DoDRM3*, on average being enhanced 3.79-fold.

**Fig. 10:**
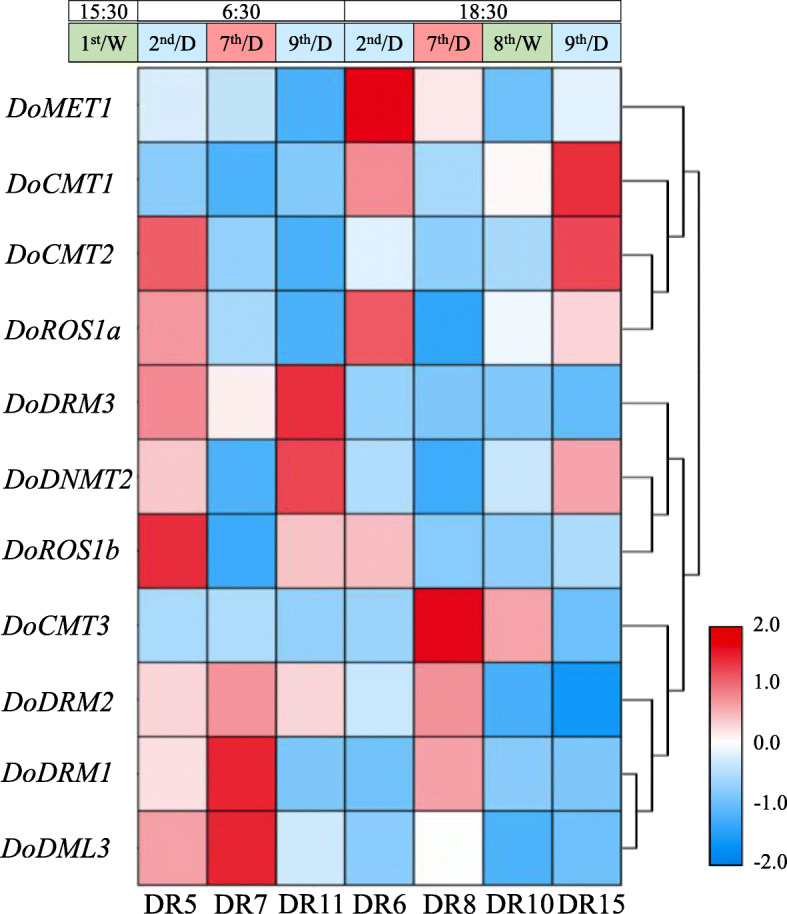
Transcript levels of *DoC5-MTase* and *DodMTase* genes in response to drought stress. Heat map was generated by log2 transformation of the average of the expression values of *D. officinale DoC5-MTase* and *DodMTase* genes subjected to drought treatment. *D. officinale* seedlings were irrigated on the 1st day, dried from the 2nd to the 7th day, and re-watered on the 8th day as shown in Fig. S10. Samples were collected at different times, DR5/DR6, DR7/DR8, and DR11/DR15 were picked at 06:30 and 18:30 on the 2nd, 7th and 9th day, respectively, and DR10 was harvested at 18:30 on the 8th day. Blue and red in the color scale indicate low and high transcript expression, respectively

To explore the potential roles of *DoC5-MTase* and *DodMTase* genes exposed to drought stress, their transcript abundance was evaluated by analyzing the RNA-seq data [32] under different drought treatments. As shown in Fig. 10, *DoMET1* was downregulated at both dawn (− 1.04-fold) and dusk (− 1.32-fold) during drought for 7 days. *DoDRM3* was also downregulated at both dawn (− 1.14-fold) and dusk (− 1.05-fold) during drought for 7 days, but the down-regulation (without a fold-change ≤ − 1.2) was not significant. Transcript levels of *DoDML3* and *DoROS1b* remained high (1.56- to 52.36-fold compared to other genes) throughout 7 days of drought stress. Over time, as drought stress increased, the expression of *DoDRM1*, *DoDRM2* and *DoCMT3* was notably enhanced at dawn (1.93-, 1.05- and 1.01-fold, respectively) throughout 7 days of drought stress, and their expression also improved at dusk (12.41-, 1.14- and 2.45-fold, respectively). Conversely, *DoCMT1*, *DoCMT2*, *DoROS1a*, *DoROS1b* and *DoDNMT2* were downregulated at dawn (− 1.45, − 1.71, − 1.24, − 2.66, and − 1.26-fold, respectively) during drought for 7 days, and their expression was also repressed at dusk (− 1.88, − 1.23, − 1.56, − 1.58, and − 1.13-fold, respectively). The expression of *DoDNMT2* was upregulated 1.42-fold after the rewatering treatment. Overall, the expression levels of *DoC5-MTase* and *DodMTase* genes were significantly influenced by cold and drought stress.

### Transcription levels of *DoC5-MTase* and *DodMTase* genes during the accumulation of *D. officinale* polysaccharides

**Fig. 11:**
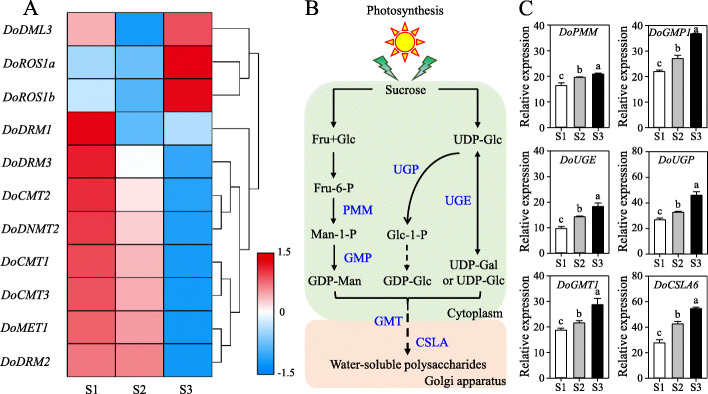
Transcript levels of *DoC5-MTase* and *DodMTase* genes, as well as the genes involved in the biosynthesis of WSPs, at different developmental stages. **a** The expression patterns of *DoC5-MTase* and *DodMTase* genes during different developmental stages. **b** Proposed biosynthetic pathway of WSPs in *D. officinale*. **c** The expression patterns of genes involved in the biosynthesis of WSPs during different developmental stages. The three developmental stages are protocorm-like bodies (S1), shoots (S2) and plantlets (derived from protocorm-like bodies, S3), which were collected at 2, 4 and 10 months after germination, respectively. The heat map was generated by log2 transformation of the average of the expression values of *D. officinale DoC5-MTase* and *DodMTase* genes during different developmental stages. Blue and red in the color scale indicate low and high transcript expression, respectively. In C, error bars indicate the mean ± SD (standard deviation) of three individual experiments, and were quantified with three independent replicates. Different lowercase letters above bars indicate a significant difference among different stages at *p* < 0.05 based on Duncan’s multiple range test. CSLA, cellulose synthase-like A; Fru, fructose; Gal, galactose; Glc, glucose; GMP, GDP-mannose pyrophosphorylase; Man, mannose; PMM, phosphomannomutase; UGE, UDP-glucose epimerase; UGP, UDP-glucose pyrophosphorylase; WSP, water-soluble polysaccharide

*D. officinale* is a precious Chinese herb with abundant secondary metabolites, especially WSPs, which account for 34.06–43.27% w/w [28], in its stems. Furthermore, WCPs in *D. officinale* accumulated at different developmental periods. To elucidate the potential roles of *DoC5-MTase* and *DodMTase* genes involved in the biosynthesis of WSPs, the transcription levels of these genes were assessed across three developmental periods (Fig. 11a). The expression of *DodMTase* genes (*DoDML3*, *DoROS1a* and *DoROS1b*) was significantly upregulated during a range of plant growth stages (from protocorm-like bodies to plantlets), corresponding to a gradual increase in WSP content that ranged from 93.71 to 183.74 mg g^− 1^ (Figure S11D), as well as the upregulation of six key genes (*DoPMM*, *DoGMP1*, *DoUGP*, *DoUGE*, *DoGMT1* and *DoCSLA6*) involved in the biosynthesis of WSPs (Fig. 11c). In contrast, in the same stages, the transcript levels of all *DoC5-MTase* genes (*DoMET1*, *DoCMT1*, *DoCMT2*, *DoCMT3*, *DoDRM1*, *DoDRM2*, *DoDRM3* and *DoDNMT2*) were markedly reduced. Furthermore, the transcript levels of *DodMTase* genes (*DoDML3*, *DoROS1a* and *DoROS1b*) were positively correlated with WSP content (Pearson’s correlation coefficient *R*^*2*^ = 0.57, 0.87 and 0.79, respectively) during three developmental stages (protocorm-like bodies, shoots and plantlets). In contrast, the expression of *DoC5-MTase* genes (*DoMET1*, *DoCMT1*, *DoCMT2*, *DoCMT3*, *DoDRM1*, *DoDRM2*, *DoDRM3* and *DoDNMT2*) were negatively correlated with WSP content (Pearson’s correlation coefficient *R*^*2*^ = − 0.94, − 0.95, − 0.83, − 0.92, − 0.66, − 0.90, − 0.96, and − 0.86, respectively). These findings suggest that *DoC5-MTase* and *DodMTase* genes together play roles in the accumulation of WSPs.

## Discussion

*D. officinale* is a traditional Chinese herb that has been used extensively as a tonic and precious food for thousands of years, due to its abundant bioactive constituents, such as polysaccharides, alkaloids, flavones, and amino acids [27, 38]. Many genes involved in the biosynthesis of bioactive secondary metabolites have been discovered and identified in this orchid. However, no information is available about epigenetic factors (DNA methylation and demethylation) regulating gene expression during the formation of secondary metabolites in *D. officinale*. To elucidate the regulatory role of DNA methylation and demethylation in *D. officinale*, we identified the DoC5-MTase and DodMTase genes at the whole-genome level. The *cis*-acting elements, conserved motifs, phylogenetic relationships, protein-protein interactions, and sequence features of these genes were also integratively analyzed.

According to the *D. officinale* reference genome [33, 34], a total of eight DoC5-MTase and three DodMTase genes were discovered and identified (Table 1). However, the number of C5-MTase and dMTase gene families mined in *A. thaliana* (15), globe artichoke (*Cynara cardunculus*) (17) and *S. miltiorrhiza* (14) is higher than that of *D. officinale* (11). Since the *D. officinale* genome (1.35 GB) is approximately 2- to 11-fold larger than that of *A. thaliana* (125 MB) [39], globe artichoke (725 MB) [40] and *S. miltiorrhiza* (558 MB) [41], this suggests that DoC5-MTase and DodMTase may have gene absence or mutation events in *D. officinale*. Moreover, *A. thaliana* contains four MET genes, while *D. officinale* has only one (Figs. 4, 5), indicating the loss of MET genes during the evolution of *D. officinale*. Similarly, no DME was found in *O. sativa* or *D. officinale*, unlike in *A. thaliana* (Figs. 4, 5). Although ROS1 was found in *D. officinale*, it contains two paralogs, DoROS1a and DoROS1b (Figs. 4, 5), inferring the occurrence of ROS1 gene duplication. In previous studies, 756 gene families expanded, whereas 804 gene families contracted during the evolution of *D. officinale* [34]. The methylation-related genes are associated with whole genome duplication and tandem replication, along with gene absence events [42, 43], thus resulting in the differential gene number between *D. officinale* and other plant species, such as *A. thaliana*, globe artichoke and *S. miltiorrhiza*. Gene duplication and loss events play a pivotal role in the evolution of *DoC5-MTase* and *DodMTase* gene families, which is in agreement with research in *Fragaria vesca* [44] and tea plant [26]. Taken together, the duplication of *DoROS1* and the loss of *DoDME* and *DoMET1* implied that *DoC5-MTase* and *DodMTase* might have functional redundancy and divergence.

Phylogenetic analysis illustrated that DoC5-MTase was divided into four classes, including MET, CMT, DRM and DNMT (Figs. 3, 4, 5), which are highly conserved in both dicots and monocots, such as *A. thaliana* [21] and *O. sativa* [45]. DodMTase was grouped into two categories, DML3 and ROS1 (Fig. 2), which is inconsistent with the result of dicots, such as *A. thaliana* [21] and peanut (*Arachis hypogaea*) [22], but is consistent with the results of monocots, such as rice and maize [46]. This suggests that DME may be phylogenetically monophyletic in dicots. Furthermore, each C5-MTase or dMTase subfamily was further divided into dicot and monocot groups (Fig. 5). These results were verified by MEME analysis, and the conserved motifs were similar within each sub-cluster, but divergent among different clusters (Fig. 5), indicating that dicots and monocots may adopt different tactics in the evolution of C5-MTases and dMTases.

Within a gene family, genes harboring the same domains and motifs may perform similar roles. Cytosine-5 DNA methylation, including de novo and maintenance DNA methylation, is antagonistically controlled by C5-MTases and dMTases. CMT, DRM, and MET are responsible for the maintenance of CHG, CHH, and CG methylation, respectively, while DRM is the main de novo C5-MTase [1, 2, 4]. DNMT2 exhibits transfer RNA methyltransferase activity [47], but its role in cytosine-5 DNA methylation is still unknown. DNA methylation plays a critical role in growth and development. The *drm1drm2cmt3* triple mutation leads to a dwarf phenotype, partial sterility, and slow growth in *A. thaliana* [48]. DNA methylation is also implicated in the regulation of fruit ripening. Genes involved in RNA-directed DNA methylation, including *FvCMT3.1*, *FvCMT3.2*, *FvDRM1.3*, and *FvDRM3.1*, are downregulated and thus lead to DNA hypomethylation during strawberry ripening [49]. For DNA demethylase, *ROS1* dysfunction can silence the expression of the *RD29A* gene, because of its hypermethylation at the *RD29A* promoter [50]. DME activates maternal *FIS2*, *FWA*, and *MEA* allele expression, and is responsible for endosperm gene imprinting and seed viability [51]. *SlDML2*, a *ROS1* ortholog, is required for tomato fruit ripening, by activating DNA demethylation and promoting the expression of fruit-ripening genes, such as *CNR*, *PSY1*, and *RIN* [13]. *OsROS1a* plays an irreplaceable role in both male and female gametophytes [52], while *OsROS1c* enhances expression and transposition of *Tos17* in rice callus [53]. This suggests that *C5-MTase* and *dMTase* genes from a cluster might also participate in distinct biological processes.

Increasing evidence shows that C5-MTases and dMTases are involved in abiotic stress responses [7–9]. In the present study, a large amount of hormone-, light-, and stress-responsive *cis*-acting elements was detected in the promoter regions of *DoC5-MTase* and *DodMTase* genes (Fig. 7). Besides, *cis*-acting elements function as molecular switches, participating in the transcriptional regulation of stress-inducible gene expression and regulating various biological processes [54]. In addition, abiotic stress can cause variation in cytosine DNA methylation by generating novel epialleles, potentially affecting transcript abundance [55]. This effect may account for the significant changes in the transcript abundance of *DoC5-MTase* and *DodMTase* under cold stress (Fig. 9), and drought stress (Fig. 10). Consistent with a previous study [56], *cis*-acting elements harboring stress-inducible roles in *DoC5-MTase* and *DodMTase*, may respond to multiple stress signals and affect gene expression.

Notably, DNA methylation and demethylation are involved in the regulation of secondary metabolism. WSPs are important secondary metabolites and account for the commercial quality of *D. officinale* [33]. The WSP content in different parts of *D. officinale* differs (stem > leaves > roots) [38]. Herein, *DoC5-MTase* and *DodMTase* genes exhibited significantly differential transcription levels in the flower bud, gynostemium, labellum, leaf, pollinium, root, sepal, and stem (Fig. 8). The trend of *DoROS1a* expression is consistent with that of the WSP content, whereas the trend of *DoCMT3* expression was contrary to the above, indicating that *DoC5-MTase* and *DodMTase* are associated with the production of WSPs. In addition, cold stress can markedly improve WSP content in *D. officinale* [31, 37]. Interestingly, three *DodMTase* genes was upregulated, and eight *DoC5-MTase* genes were downregulated (Fig. 9) either in response to cold stress or during seedling development (Fig. 11). DNA methylation inhibits gene transcription. In contrast, DNA demethylation enhances gene transcription. Cold treatment may activate the enzymes involved in the biosynthesis of WSPs through increased DNA demethylation and decreased DNA methylation. Taken together, DoC5-MTase and DodMTase may work together, affecting the accumulation of WSPs by regulating the level of DNA methylation, but the mechanisms need to be studied in greater detail.

## Conclusions

According to the *D. officinale* reference genome, eight *DoC5-MTase* genes and three *DodMTase* genes were discovered and identified. Phylogenetic analysis demonstrated that the eight *DoC5-MTase* genes were divided into four categories, *DoCMT*, *DoDRM*, *DoMET*, and *DoDNMT*. The three *DodMTase* genes were grouped into *DoROS* and *DoDML* subfamilies. The expression profiles of *DoC5-MTase* and *DodMTase* genes suggest their functional importance in the accumulation of WSPs and response to stresses in *D. officinale*. These findings will be helpful to reveal the possible roles of DoC5-MTase and DodMTase in secondary metabolism and stress responses in *D. officinale*, and provide constructive clues for further exploration of the epigenetic mechanism of DoC5-MTase and DodMTase in orchids.

## Methods

### Plant material and experimental treatments

*D. officinale* “Zhongke 1” (a high-WSP line, 49.5% w/w; genetic breeding by Prof. Jun Duan at the South China Botanical Garden, Chinese Academy of Sciences, Approval no. 20180003, website: https://www.cas.cn/syky/201810/t20181031_4668440.shtml) plants were cultivated and acclimatized in a walk-in phytotron at the South China Botanical Garden, Chinese Academy of Sciences, Guangzhou, in China. Tissue-specific mRNA expression patterns of *DoC5-MTase* and *DodMTase* genes were assessed for protocorm-like bodies, shoots and plantlets (derived from protocorm-like bodies) during the vegetative stage (2, 4 and 10 months after *D. officinale* seeds were germinated on half-strength Murashige and Skoog medium [57], respectively; Figure S10A, B, C), as well as 10 tissues, including flower buds, gynostemium (column), sepals, labellum (lip), leaves, roots, stems, pollinia, white part of roots and green root tips at the reproductive stage (14 months after seedlings were transplanted into plastic pots; Fig. 8a). Four-month-old seedlings were placed in controlled-climate chambers with a 12-h light/12-h dark cycle, 80% relative humidity, and 60 μmol m^− 2^ s^− 1^ photosynthetically active radiation at 0 °C (for cold treatment) and 20 °C (for ambient control) for 20 h, respectively. All sampled materials were free of any insects, disease or mechanical damage. Each treatment was conducted as three replications and with at least 10 independent plants per condition. All these samples were immediately stored at − 80 °C after freezing in liquid nitrogen.

### Identification of the cytosine-5 DNA methyltransferase and demethylase genes

The *D. officinale* genome was retrieved from the online Herbal Medicine Omics Database (http://herbalplant.ynau.edu.cn/) [33], or downloaded from DDBJ/EMBL/GenBank under the accession code SUB764497 [34]. The Hidden Markov model (HMM) of cytosine-5-specific DNA-methylase (PF00145), was obtained from the PFAM database (http://pfam.xfam.org/) and employed C5-MTase proteins with HMMER software version 3.2.1 (http://hmmer.org/). Similarly, to search the dMTase proteins, the HMMs of the helix-hairpin-helix, the Gly/Pro-rich loop (HhH-GPD, PF00730) and RNA recognition motif demethylase (RRM-DME, PF15628) were downloaded from the PFAM database as the probes. Afterwards, C5-MTase and dMTase proteins from *D. officinale* were verified via a local HMM-based search program (E-value ≤ 1e^− 10^). Subsequently, these candidate proteins, annotated as “cytosine-5 DNA methyltransferase” or “DNA demethylase” with the best “C5-MTase” or “HhH-GPD and RRM-DME” hits, were retained for further confirmation with the Conserved Domain Database (NCBI-CDD, https://www.ncbi.nlm.nih.gov/cdd) and the Simple Modular Architecture Research Tool (SMART, http://smart.embl-heidelberg.de/). Redundant sequences and incomplete proteins containing no characteristic PF00145, PF00730 or PF15628 motifs were manually removed. All putative methyltransferase and demethylase genes that were identified are listed in Table S1. The grand average of hydrophobicity (GRAVY), molecular weight (MW) and isoelectric point (pI) of DoC5-MTase and DodMTase proteins were calculated using the ExPASy tool (http://web.expasy.org/protparam). Plant-mPLoc (http://www.csbio.sjtu.edu.cn/bioinf/plant-multi/) was used to predict the sub-cellular localization of *DoC5-MTase* and *DodMTase* genes.

### Phylogenetic tree construction

DNAMAN version 6.0 software (Lynnon Biosoft, Foster City, CA, USA) was used to generate multiple sequence alignments of the C5-MTase and dMTase proteins among different plant species including *A. lyrata*, *A. thaliana*, *Brachypodium distachyon*, *Citrus clementina*, *Cucumis sativus*, *Erythranthe guttata*, *Glycine max*, *Manihot esculenta*, *Oryza sativa*, *Populus trichocarpa*, *R. communis*, *S. lycopersicum*, *Salvia miltiorrhiza*, *Sorghum bicolor*, *Zea mays* and *Vitis vinifera*, that were retrieved from Phytozome version 12.1.6 (https://phytozome.jgi.doe.gov/pz/portal.html#). To verify the sequence alignments using DNAMAN, multiple alignment of the C5-MTase and dMTase proteins was also performed using the ClustalX 2.1 program [58]. The resulting file was used to build a phylogenetic tree with the Molecular Evolutionary Genetics Analysis (MEGA X) software [59] based on the neighbor-joining method [60] with 1000 bootstrap replicates. DoC5-MTase and DodMTase were classified based on their phylogenetic relationship with the corresponding *A. thaliana* C5-MTase and dMTase proteins.

### Analysis of conserved motifs, gene structure and protein-protein interaction

Conserved motifs of all DoC5-MTase and DodMTase proteins were analyzed using Multiple Expectation Maximization for Motif Elicitation (MEME, http://meme-suite.org/) software version 5.0.5 by uploading the amino acid sequences following the MEME instructions. All coding sequences and genomic sequences of *DoC5-MTase* and *DodMTase* genes were used as individual queries to visualize the schematic diagrams of the genes’ structures using Gene Structure Display Server version 2.0 (GSDS, http://gsds.cbi.pku.edu.cn/). The STRING 11 tool (https://string-db.org) was used to construct a protein-protein interaction network.

### RNA preparation and RT-qPCR analysis

Total RNA was extracted from the flowers, leaves, roots and stems of 14-month-old *D. officinale* after transplantation by RNAout2.0 reagent (Tiandz Inc., Beijing, China) as described previously [25]. RNA was purified with RNase-free DNase I (Takara Bio Inc., Kyoto, Japan). First-strand cDNA was synthesized from 1 μg of total RNA using the PrimeScript Reagent Kit with gDNA Eraser (Takara Bio Inc.) according to the supplier’s instructions. The resulting cDNA was diluted 1:50 and used as a template for RT-qPCR analysis. RT-qPCR was performed on a LightCycler 480 instrument (Roche Diagnostics, Mannheim, Germany) on 96-well plates with iTaq™ Universal SYBR® Green Supermix (Bio-Rad Laboratories, Hercules, CA, USA) with an identified amplification program for 40 cycles, as described previously [28]. The constitutively expressed *D. officinale ACTIN* (NCBI accession number: JX294908) was used as the internal standard for normalizing variations in cDNA concentration. In addition, at least three biological replicates derived from at least three independently generated cDNA samples were analyzed. The specific primer sequences of the *C5-MTase* and *dMTase* genes are listed in Table S1.

### Transcriptome analysis of *DoC5-MTase* and *DodMTase* gene expression

The expression profiles of *DoC5-MTase* and *DodMTase* genes under ambient (20 °C) and cold (0 °C) for 20 h were investigated [34]. RNA-seq reads were obtained from the NCBI sequence read archive (SRA) database under accession numbers SRR3210613, SRR3210621, SRR3210626, SRR3210630, SRR3210635 and SRR3210636. To detect the transcription abundance of *DoC5-MTase* and *DodMTase* genes subjected to drought and re-watering treatments, *D. officinale* seedlings were watered on the 1st day, and kept unwatered to mimic natural drought stress from the 2nd to the 7th day, and rewatered on the 8th day. Samples were individually collected at both 06:30 and 18:30 on the 2nd (DR5 and DR6), 7th (DR7 and DR8), and 9th (DR11 and DR15) day, and at 18:30 on the 8th day (DR10). The RNA-seq data of DR5 (SRR7223299), DR6 (SRR7223298), DR7 (SRR7223301), DR8 (SRR7223300), DR10 (SRR7223296), DR11 (SRR7223295) and DR15 (SRR7223297) were downloaded from the NCBI SRA provided by Wan et al. [32]. All clean reads were aligned to the *D. officinale* reference genome [33, 34] using HISAT2 software [61] with defaults parameters. After the final transcriptome was established, StringTie software [62] was used to estimate the differential transcript levels for mRNAs by quantifying the FPKM value. The expression levels of all transcripts were indicated by calculating fragments per kilobase per million (FPKM). The differentially expressed *DoC5-MTase* and *DodMTase* genes were defined with log2 (fold change) > 1 or < − 1 and with statistical significance (*p* < 0.05). Furthermore, the targeted genes with a fold-change ≥ 1.2 were defined as upregulated genes, and those with a fold-change ≤ − 1.2 were regarded as down-regulated genes.

### Determination of WSP content in *D. officinale* during three developmental periods

The WSP content was quantified using the phenol-sulfuric acid method as described previously [31]. Briefly, powdered samples (0.3 g) of *D. officinale* protocorm-like bodies, shoots and plantlets was pre-extracted with aqueous-ethanol solution (80%, v/v) at 80 °C for 2 h, to remove small molecular weight compounds. The residue was re-extracted with 100 mL of distilled water at 100 °C for 2 h, then filtered through Whatman No. 1 filter paper (Congyuan Instrument Co., Guangzhou, China). The filtrate was used to assess the WSP content with a phenol-sulfuric acid method based on a glucose standard curve at 488 nm on a UV-6000 spectrophotometer (Shanghai Metash Instruments Co., Shanghai, China).

### Statistical analysis

All data were expressed as the mean ± standard error of more than three independent biological replicates for each determination. Statistical analysis was performed using IBM SPSS version 22.0 for Windows (IBM Corp., Armonk, NY, USA). The significant difference of transcript levels between CK and CA at *p* < 0.01 was analyzed based on the Student’s *t*-test. Unless otherwise indicated, all data were compared by using analysis of variance (ANOVA) followed by Duncan’s multiple range test at *p* < 0.05. Heat maps were generated by the software TBtools (https://github.com/CJ-Chen/TBtools), and their color scales represent the log2-transformation of the average of expression values, with low expression in blue and high expression in red. Correlation analysis was performed using Pearson’s correlation coefficient (*R*^*2*^) at *p* < 0.01.

## Supplementary Information

**Additional file 1: Table S1**. Primers used for RT-qPCR analysis in this study. Gene-specific primers for real-time reverse transcription quantitative PCR (RT-qPCR) were designed by the PrimerQuest tool (http://www.idtdna.com/Primerquest/Home/Index). The *D. officinale ACTIN* gene was obtained from NCBI (GenBank accession no. JX294908). F, forward; R, reverse

**Additional file 2: Table S2**. Distribution of conserved motifs in DoC5-MTase based on the results of MEME (http://meme-suite.org/) analysis

**Additional file 3: Table S3**. Distribution of conserved motifs in DodMTase based on the results of MEME (http://meme-suite.org/) analysis

**Additional file 4: Table S4**. Information of C5-MTase genes in the 11 tested species

**Additional file 5: Table S5**. Information of dMTase genes in the 16 tested species

**Additional file 6: Table S6**. Number of *cis*-elements in the promoter region of *DoMTase* and *dMTase* genes

**Additional file 7: Figure S1**. Sequence alignment of MET1 protein sequences from *D. officinale* and *A. thaliana*

**Additional file 8: Figure S2**. Sequence alignment of CMT protein sequences from *D. officinale* and *A. thaliana*

**Additional file 9: Figure S3**. Sequence alignment of DRM protein sequences from *D. officinale* and *A. thaliana*

**Additional file 10: Figure S4**. Sequence alignment of DNMT2 protein sequences from *D. officinale* and *A. thaliana*

**Additional file 11: Figure S5**. Sequence alignment of DML3 protein sequences from *D. officinale* and *A. thaliana*

**Additional file 12: Figure S6**. Sequence alignment of ROS1 protein sequences from *D. officinale* and *A. thaliana*

**Additional file 13: Figure S7**. Distribution of conserved motifs in DoC5-MTase based on the results of MEME analysis

**Additional file 14: Figure S8**. Distribution of conserved motifs in DodMTase based on the results of MEME analysis

**Additional file 15: Figure S9**. Number of *cis*-elements in the promoter region of *DoC5-MTase* and *DodMTase* genes

**Additional file 16: Figure S10**. The experimental scheme of *D. officinale* seedlings under sustained drought treatment and irrigation. Green arrows at 15:30 indicate the irrigation times, and magenta arrows indicate the experimental sampling times. White bars indicate light periods, and black bars indicate dark periods

**Additional file 17: Figure S11**. WSP content in juvenile *D. officinale* stems at three different developmental stages (protocorm-like bodies, shoots and plantlets). (A-C) Three developmental stages (protocorm-like bodies, shoots and plantlets, namely S1, S2 and S3, respectively), which correspond to 2, 4 and 10 months after germination, respectively. (D) WSP content in protocorm-like bodies, shoots and plantlets of juvenile *D. officinale* stems. Error bars indicate the mean ± SD (standard deviation) of three individual experiments, and were performed in triplicate. Different letters above bars indicate a significant difference among different stages at *p* < 0.05 based on Duncan’s multiple range test. DW, dry weight. WSP, water-soluble polysaccharide

## Data Availability

The datasets supporting the conclusions of this article are included within the article and its additional files. All data and plant materials used in current study are available from the corresponding author on reasonable request.

## Abbreviations

C5-MTase: Cytosine-5 DNA methyltransferase
CMT3: CHROMOMETHYLASE3
DME: DEMETER
DML2: DEMETER-like 2
DML3: DEMETER-like 3
dMTase: DNA demethylase
DRM2: DOMAINS REARRANGED METHYLASE2
FPKM: Fragments per kilobase per million
GRAVY: Grand average of hydrophobicity
HMM: Hidden Markov model
MEGA: Molecular evolutionary genetics analysis
MEME: Multiple expectation maximization for motif elicitation
MET1: METHYLTRANSFERASE1
MW: Molecular weight
ORF: Open reading frame
pI: isoelectric point
ROS1: REPRESSOR OF SILENCING1
RT-qPCR: Reverse transcription quantitative polymerase chain reaction
WSP: Water-soluble polysaccharide
