# Supplementary material for: Genome-wide identification and analysis of DNA methyltransferase and demethylase gene families in Dendrobium officinale reveal their potential functions in polysaccharide accumulation
Source: BMC Plant Biol. 2021 Jan 6;21:21. doi: 10.1186/s12870-020-02811-8 (PMC7789594; doi:10.1186/s12870-020-02811-8)
Supplement: Supplementary file 1 — Additional file 1: Table S1. Primers used for RT-qPCR analysis in this study. Gene-specific primers for real-time reverse transcription quantitative PCR (RT-qPCR) were designed by the PrimerQuest tool (http://www.idtdna.com/Primerquest/Home/Index). The D. officinale ACTIN gene was obtained from NCBI (GenBank accession no. JX294908). F, forward; R, reverse [file 12870_2020_2811_MOESM1_ESM.pdf]

Supplemental Table S1. Primers used for RT-qPCR analysis in this study.

Primers	Primer sequences (5′→3′)
DoMET1F	GTTGGTGGAGTTGTGGTTATTG
DoMET1R	GTTCCATCAAGCCTCTCGTATAA
DoCMT1F	GTTGCTGTACCAGTTTCAAGAG
DoCMT1R	GGAAGTTTGCAGGAAGAGTAAAG
DoCMT2F	GTATGCCTTGAGTCGTTTGATTG
DoCMT2R	AAGACACGCAAGCGAAATTG
DoCMT3F	GCGTGTCTGTAGAGTTCCTAAA
DoCMT3R	TCCACCTCTGGATCAAATTCC
DoDRM1F	CATACTAGAGGTGGAGGCATTG
DoDRM1R	TCAGCACTGAAAGGTGGTAAG
DoDRM2F	GCATCAACTTGCTTTCTCTCTTC
DoDRM2R	ACCTCTGATTTCTCAACCGATAC
DoDRM3F	CACTACGGGATACTTGCTCTTC
DoDRM3R	CCCTGTCCAACCCAGATTATAG
DoDNMT2F	CTGGCAAAGAGAAAGCCAATATC
DoDNMT2R	ATGCATCCATCAGCGTCTT
DoDML3F	GAGAGGAACCGTGAGAGTTTG
DoDML3R	TCATCCCTCGCTCTCGAATA
DoROS1aF	GAGCTCCTGTTCTAGACGAATG
DoROS1aR	TACTAAGGTCAGAGACGGAGAC
DoROS1bF	TGATGCGTCCGTCTCAAATC
DoROS1bR	CGTCAGATAGAACAGCCAAGTC
DoActinF	TCCCAAGGCAAACAGAGAAA
DoActinR	GGCCACTAGCATATAGGGAAAG

Gene-specific primers for real-time reverse transcription quantitative PCR (RT-qPCR) were designed by the PrimerQuest tool (http://www.idtdna.com/Primerquest/Home/Index). The *D. officinale* actin gene (*DoActin*) was obtained from NCBI (GenBank accession no. JX294908). F, forward; R, reverse.
