# Supplementary material for: Genome-wide identification and analysis of DNA methyltransferase and demethylase gene families in Dendrobium officinale reveal their potential functions in polysaccharide accumulation
Source: BMC Plant Biol. 2021 Jan 6;21:21. doi: 10.1186/s12870-020-02811-8 (PMC7789594; doi:10.1186/s12870-020-02811-8)
Supplement: Supplementary file 2 — Additional file 2: Table S2. Distribution of conserved motifs in DoC5-MTase based on the results of MEME (http://meme-suite.org/) analysis [file 12870_2020_2811_MOESM2_ESM.pdf]

Supplemental Table S2. Distribution of conserved motifs in DoC5-MTase based on the results of MEME (http://meme-suite.org/) analysis.

Name	Sequences	Width	Sites	E-value
Motif 1	ALASLLEMGYQVRLGIMEAGCYGLPQFRKRVFJWAAAPSEVLPZFPLPTH	50	16	$3.4\times e^{-476}$
Motif 2	HPEQDRILTVRECARLQGFPDSYKFAGNIKEKYRQIGNAVAVPVARALGY	50	15	$8.2\times e^{-469}$
Motif 3	VDVICGGPPCQGISGMNRFRRSGWPLEDC	29	21	$2.0\times e^{-281}$
Motif 4	FLSIVDYLKPKYVLMENVVDILKFNKGQL	29	16	$3.0\times e^{-213}$
Motif 5	HGKSKGPFGRLWWDENVPTVVTDPZPHGQ	29	15	$4.0\times e^{-208}$
Motif 6	NSFQVDTVAYHLSVLKSJFPNGJNVLSLFSGIGGAEVALHRLGIHLKTVV	50	8	$2.6\times e^{-198}$
Motif 7	GPPFFYYENVALAPKGVWTTISRFLYDIEPEFVDSKYFCAA	41	8	$8.8\times e^{-140}$
Motif 8	LYDHIPLKLNEDBLIRVKRIPKRKGABFRDLPGVIV	36	15	$9.3\times e^{-254}$
Motif 9	NLVTKWAVDYNEFACQSLKLNHPETQVRNEKAEDFLRL	38	12	$6.3\times e^{-164}$
Motif 10	RKRGYIHNLPIENRFPILPPPKKTIQEALPLTKRWWPSWDKRTQLNCLQT	50	7	$2.2\times e^{-149}$
