# Supplementary material for: Genome-wide identification and analysis of DNA methyltransferase and demethylase gene families in Dendrobium officinale reveal their potential functions in polysaccharide accumulation
Source: BMC Plant Biol. 2021 Jan 6;21:21. doi: 10.1186/s12870-020-02811-8 (PMC7789594; doi:10.1186/s12870-020-02811-8)
Supplement: Supplementary file 3 — Additional file 3: Table S3. Distribution of conserved motifs in DodMTase based on the results of MEME (http://meme-suite.org/) analysis [file 12870_2020_2811_MOESM3_ESM.pdf]

Supplemental Table S3. Distribution of conserved motifs in DodMTase based on the results of MEME (http://meme-suite.org/) analysis.

Name	Sequences	Width	Sites	E-value
Motif 1	KLDQRTLYELHYQMITFGKVFCTKSKPNCNACPMRGECKHF	41	13	$1.8\times e^{-388}$
Motif 2	FIARMHLVQGDRRFSPWKGSVVDSVVGVFLTQNVSDHLSSSAFMALAAKF	50	13	$1.8\times e^{-435}$
Motif 3	AFPVDTNVGRICVRLGWVPLQPLPESLQLHLLELYPVLESIQKYLWPRLC	50	12	$1.1\times e^{-424}$
Motif 4	HGSIDLEWLRDVPPDKAKDYLLSIRGLGLKSVECVRLLTLH	41	12	$7.0\times e^{-300}$
Motif 5	VRGTJLIPCRTAMRGSFPLNGTYFQVNEVFADHESSLNPIB	41	10	$8.2\times e^{-271}$
Motif 6	LTTEEIQHCFWRGFVCVRGFDRKTRAPKPLYARLHF	36	12	$1.5\times e^{-209}$
Motif 7	AASIPTPKLKNISRLRTEHHVYELPDSHPLLEG	33	12	$1.8\times e^{-181}$
Motif 8	ERSEBTMDSVDWEAVRCADVSEIAETIRERGMNNVLAERIKDFLNRLVRD	50	12	$7.3\times e^{-226}$
Motif 9	IWNLPRRTVYFGTSIPTIFKG	21	10	$6.6\times e^{-109}$
Motif 10	EPDDPSPYLLAIWTPGETANSIZPPKSCC	29	9	$3.2\times e^{-100}$
Motif 11	FHLVKKKRPRAKVDLDPETTRVWKLLMG	28	11	$3.7\times e^{-088}$
Motif 12	DVDKEKWWEEERRVFQGRADS	21	12	$1.1\times e^{-087}$
Motif 13	FYEDPDEIPTIKLNMEEFTQN	21	10	$1.5\times e^{-075}$
Motif 14	ASAFASARLALPSPEEKSLVP	21	10	$5.7\times e^{-057}$
Motif 15	NCEPIIEEPASPEPECPE	18	12	$2.3\times e^{-054}$
