# Supplementary material for: Genome-wide identification and analysis of DNA methyltransferase and demethylase gene families in Dendrobium officinale reveal their potential functions in polysaccharide accumulation
Source: BMC Plant Biol. 2021 Jan 6;21:21. doi: 10.1186/s12870-020-02811-8 (PMC7789594; doi:10.1186/s12870-020-02811-8)
Supplement: Supplementary file 4 — Additional file 4: Table S4. Information of C5-MTase genes in the 11 tested species [file 12870_2020_2811_MOESM4_ESM.pdf]

Supplemental Table S4. Information of *C5-MTase* genes in the 11 tested species.

Latin name of specie	Gene name	Gene ID
Arabidopsis lyrata	*AlMET1*	AL8G22690
	AlMET2	AL7G42360
	AlMET3	AL7G42980
	AlCMT3	AL2G28960
	AlCMT1	AL2G40680
	AlCMT2	AL7G35120
	AlDRM1	AL6G26240
	AlDRM2	AL6G25400
	AlDRM3	AL3G30360
	AlDNMT2	AL6G37280
Arabidopsis thaliana	*AtMET1*	AT5G49160
	AtMET2a	AT4G14140
	AtMET2b	AT4G08990
	AtMET3	AT4G13610
	AtCMT1	AT1G80740
	AtCMT2	AT4G19020
	AtCMT3	AT1G69770
	AtDRM1	AT5G15380
	AtDRM2	AT5G14620
	AtDRM3	AT3G17310
	AtDNMT2	AT5G25480
Brachypodium distachyon	*BdMET1*	Bradi1g55287
	BdMET2	Bradi1g05380
	BdCMT1	Bradi1g66167
	BdCMT3a	Bradi3g21450
	BdCMT3b	Bradi3g39050
	BdDRM1	Bradi1g77873

	BdDRM2	Bradi4g05680
	BdDRM3	Bradi2g38577
	BdDNMT2	Bradi2g43310
Erythranthe guttata	*EgMET1*	Migut.N00875
	EgCMT1	Migut.E00091
	EgCMT2a	Migut.A00577
	EgCMT2b	Migut.F00104
	EgCMT3	Migut.D02536
	EgDRM1	Migut.K01383
	EgDRM2	Migut.O00390
	EgDRM3	Migut.G00014
	EgDNMT2	Migut.E01476
Oryza sativa	*OsMET1*	LOC_Os03g58400
	OsMET2	LOC_Os07g08500
	OsCMT1	LOC_Os03g12570
	OsCMT2	LOC_Os05g13780
	OsCMT3	LOC_Os10g01570
	OsDRM1	LOC_Os11g01810
	OsDRM2	LOC_Os03g02010
Populus trichocarpa	*PtMET1*	Potri.004G134000
	PtMET2	Potri.018G138000
	PtMET3	Potri.T046100
	PtCMT2	Potri.003G100000
	PtCMT3	Potri.001G009600
	PtDRM1	Potri.001G347000
	PtDRM2	Potri.010G152200
	PtDNMT2	Potri.006G250800
Ricinus communis	*RcMET1*	29609.m000606
	RcMET2	29983.m003308

	RcCMT1	28582.m000332
	RcCMT2	29827.m002677
	RcDRM1	29631.m001043
	RcDRM2	29917.m001982
	RcDRM3	29889.m003366
	RcDNMT2	29848.m004665
Salvia miltiorrhiza	*SmMET1*	MG602207
	SmCMT1	MG602211
	SmCMT2a	MG602209
	SmCMT2b	MG602210
	SmCMT3	MG602208
	SmDRM1	MG602212
	SmDRM2	MG602213
	SmDNMT2	MG602214
Solanum lycopersicum	*SlMET1*	Solyc11g030600
	SlCMT2	Solyc12g100330
	SlCMT3	Solyc01g006100
	SlCMT4	Solyc08g005400
	SlDRM1	Solyc02g062740
	SlDRM2	Solyc04g005250
	SlDRM3	Solyc10g078190
	SlDNMT2	Solyc08g067070
Sorghum bicolor	*SbMET1*	Sobic.002G056000
	SbMET2	Sobic.001G055700
	SbCMT1	Sobic.009G083900
	SbCMT3a	Sobic.006G214000
	SbCMT3b	Sobic.004G197400
	SbDNMT2	Sobic.003G218300
	SbDRM1	Sobic.001G458100

	SbDRM2a	Sobic.001G535800
	SbDRM2b	Sobic.003G124000
	SbDRM3	Sobic.009G032200
Zea mays	*ZmMET1*	GRMZM2G334041
	ZmMET2	GRMZM2G333916
	ZmCMT1	GRMZM2G025592
	ZmCMT2	GRMZM2G005310
	ZmDRM1	GRMZM2G092497
	ZmDRM2	GRMZM2G137366
	ZmDRM3	GRMZM2G065599
	ZmDNMT2	GRMZM2G157589
