# Supplementary material for: Genome-wide identification and analysis of DNA methyltransferase and demethylase gene families in Dendrobium officinale reveal their potential functions in polysaccharide accumulation
Source: BMC Plant Biol. 2021 Jan 6;21:21. doi: 10.1186/s12870-020-02811-8 (PMC7789594; doi:10.1186/s12870-020-02811-8)
Supplement: Supplementary file 5 — Additional file 5: Table S5. Information of dMTase genes in the 16 tested species [file 12870_2020_2811_MOESM5_ESM.pdf]

Supplemental Table S5. Information of dMTase genes in the 16 tested species.

Latin name of specie	Gene name	Gene ID
Arabidopsis lyrata	*AlDME*	AL6G14210
	AlDML3	AL7G17320
	AlROS1a	AL4G32680
	AlROS1b	AL4G32670
	AlROS1c	AL3G21300
Arabidopsis thaliana	*AtDME*	AT5G04560
	AtDML2	AT3G10010
	AtDML3	AT4G34060
	AtROS1	AT2G36490
Brachypodium distachyon	*BdDME*	Bradi4g08870
	BdDML3	Bradi3g43720
	BdROS1a	Bradi2g23797
	BdROS1b	Bradi4g16620
Citrus clementina	*CcDME*	Ciclev10030474
	CcDML3	Ciclev10027676
	CcROS1	Ciclev10010892
Cucumis sativus	*CsDME*	Cucsa.308950
	CsDML3	Cucsa.385370
	CsROS1a	Cucsa.083110
	CsROS1b	Cucsa.378580
Erythranthe guttata	*EgDME1*	Migut.F00888
	EgDME2	Migut.L00609
	EgDML3	Migut.D02337
	EgROS1	Migut.J00987
Glycine max	*GmDME*	Glyma.20G188300
	GmROS1a	Glyma.03G190800
	GmROS1b	Glyma.10G065900

	GmROS1c	Glyma.13G151000
Manihot esculenta	*MeDME1*	Manes.07G091800
	MeDME2	Manes.10G055200
	MeDML3	Manes.04G008900
	MeROS1a	Manes.09G060800
	MeROS1b	Manes.08G020500
Oryza sativa	*OsDML3a*	Os02g29380
	OsDML3b	Os04g28860
	OsROS1a	Os01g11900
	OsROS1b	Os02g29230
	OsROS1c	Os05g37350
	OsROS1d	Os05g37410
Populus trichocarpa	*PtDME1*	Potri.010G234400
	PtDME2	Potri.008G025900
	PtDML3	Potri.001G150000
	PtROS1	Potri.006G116000
Ricinus communis	*RcDME*	29428.m000327
	RcDML3	29991.m000647
	RcROS1	29092.m000452
Salvia miltiorrhiza	*SmDML1*	MG602215
	SmDML2	MG602216
	SmDML3	MG602217
	SmDML4	MG602218
	SmDML5	MG602219
	SmDML6	MG602220
Solanum lycopersicum	*SlDME*	Solyc11g007580
	SlDML3	Solyc03g123440
	SlROS1a	Solyc10g083630
	SlROS1b	Solyc09g009080

Sorghum bicolor	*SbDML3*	Sobic.006G224100
	SbROS1a	Sobic.009G155900
	SbROS1b	Sobic.004G149800
	SbROS1c	Sobic.008G085300
Zea mays	*ZmDML3*	GRMZM5G828460
	ZmROS1a	GRMZM2G131756
	ZmROS1b	GRMZM2G422464
Vitis vinifera	*VvDME*	GSVIVT01034713001
	VvROS1	GSVIVT01033777001
