# Supplementary material for: Genome-wide identification and analysis of DNA methyltransferase and demethylase gene families in Dendrobium officinale reveal their potential functions in polysaccharide accumulation
Source: BMC Plant Biol. 2021 Jan 6;21:21. doi: 10.1186/s12870-020-02811-8 (PMC7789594; doi:10.1186/s12870-020-02811-8)
Supplement: Supplementary file 6 — Additional file 6: Table S6. Number of cis-elements in the promoter region of DoMTase and dMTase genes [file 12870_2020_2811_MOESM6_ESM.pdf]

Supplemental Table S6. Number of *cis*-elements in the promoter region of *DoMTase* and *dMTase* genes.

	DoMET1	*DoCMT1*	*DoCMT2*	*DoCMT3*	*DoDRM1*	*DoDRM2*	*DoDRM3*	*DoDNMT2*	*DoDML3*	*DoROS1a*	*DoROS1b*
ABA response	1	2	0	5	5	0	5	0	0	1	4
Anaerobic induction	1	1	2	3	0	3	0	1	1	3	0
Auxin response	2	0	0	2	0	3	0	2	1	0	1
Dehydration response	1	0	0	1	1	1	2	0	0	1	0
Endosperm specific	0	1	1	0	2	0	0	2	2	0	0
Ethylene response	0	1	1	0	2	0	0	2	2	2	0
GA response	1	2	2	4	2	1	4	2	1	1	0
Heat response	0	0	0	0	0	2	0	0	0	0	0
Light response	5	9	6	10	13	9	20	5	9	3	7
Low temperature response	6	1	0	2	2	2	3	1	2	2	7
MeJA response	2	2	0	4	4	4	2	0	0	0	2
Meristem specific	2	0	0	2	0	1	0	0	1	3	1
SA response	0	1	0	0	0	0	0	0	0	0	0
Stress response	3	0	3	2	2	3	1	1	3	2	4
Sucrose response	0	0	0	0	0	0	1	0	0	1	0
Sulfur response	1	0	1	1	1	6	4	1	2	5	1
Wound response	0	2	2	2	4	2	5	0	4	2	8
