# Supplementary material for: Genome-wide identification and analysis of DNA methyltransferase and demethylase gene families in Dendrobium officinale reveal their potential functions in polysaccharide accumulation
Source: BMC Plant Biol. 2021 Jan 6;21:21. doi: 10.1186/s12870-020-02811-8 (PMC7789594; doi:10.1186/s12870-020-02811-8)
Supplement: Supplementary file 11 — Additional file 11: Figure S5. Sequence alignment of DML3 protein sequences from D. officinale and A. thaliana [file 12870_2020_2811_MOESM11_ESM.pdf]

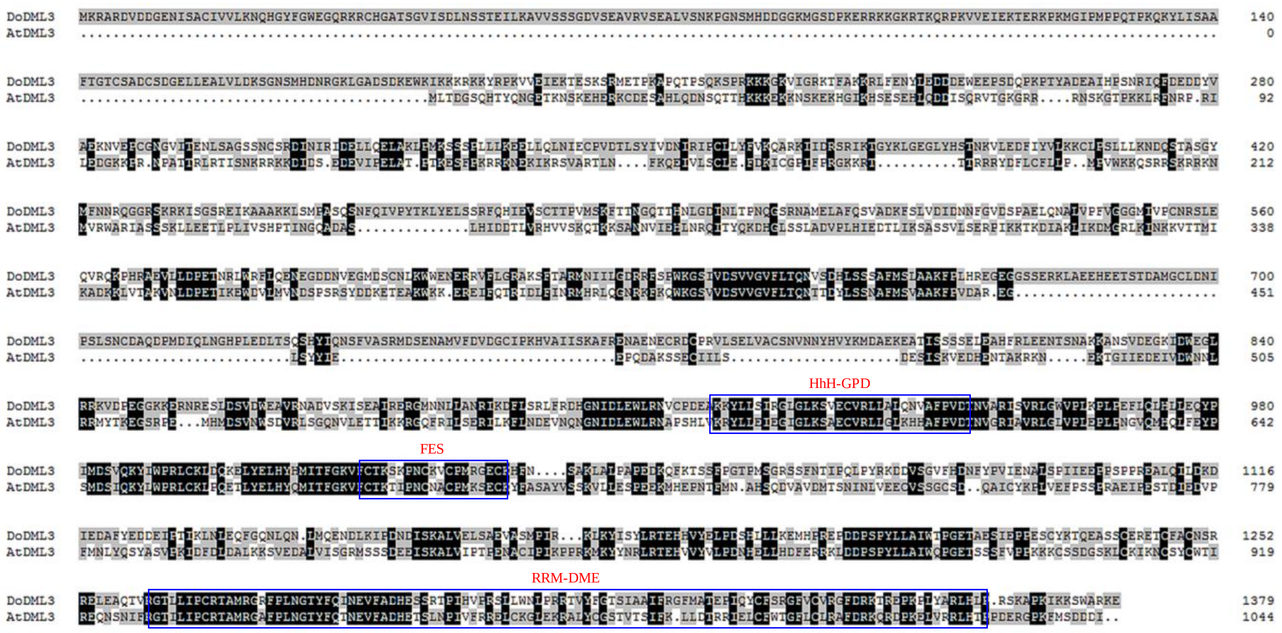


**Supplemental Figure S5. Sequence alignment of DML3 protein sequences from *D. officinale* and *A. thaliana*.**
