# Supplementary material for: Genome-wide identification and analysis of DNA methyltransferase and demethylase gene families in Dendrobium officinale reveal their potential functions in polysaccharide accumulation
Source: BMC Plant Biol. 2021 Jan 6;21:21. doi: 10.1186/s12870-020-02811-8 (PMC7789594; doi:10.1186/s12870-020-02811-8)
Supplement: Supplementary file 13 — Additional file 13: Figure S7. Distribution of conserved motifs in DoC5-MTase based on the results of MEME analysis [file 12870_2020_2811_MOESM13_ESM.pdf]

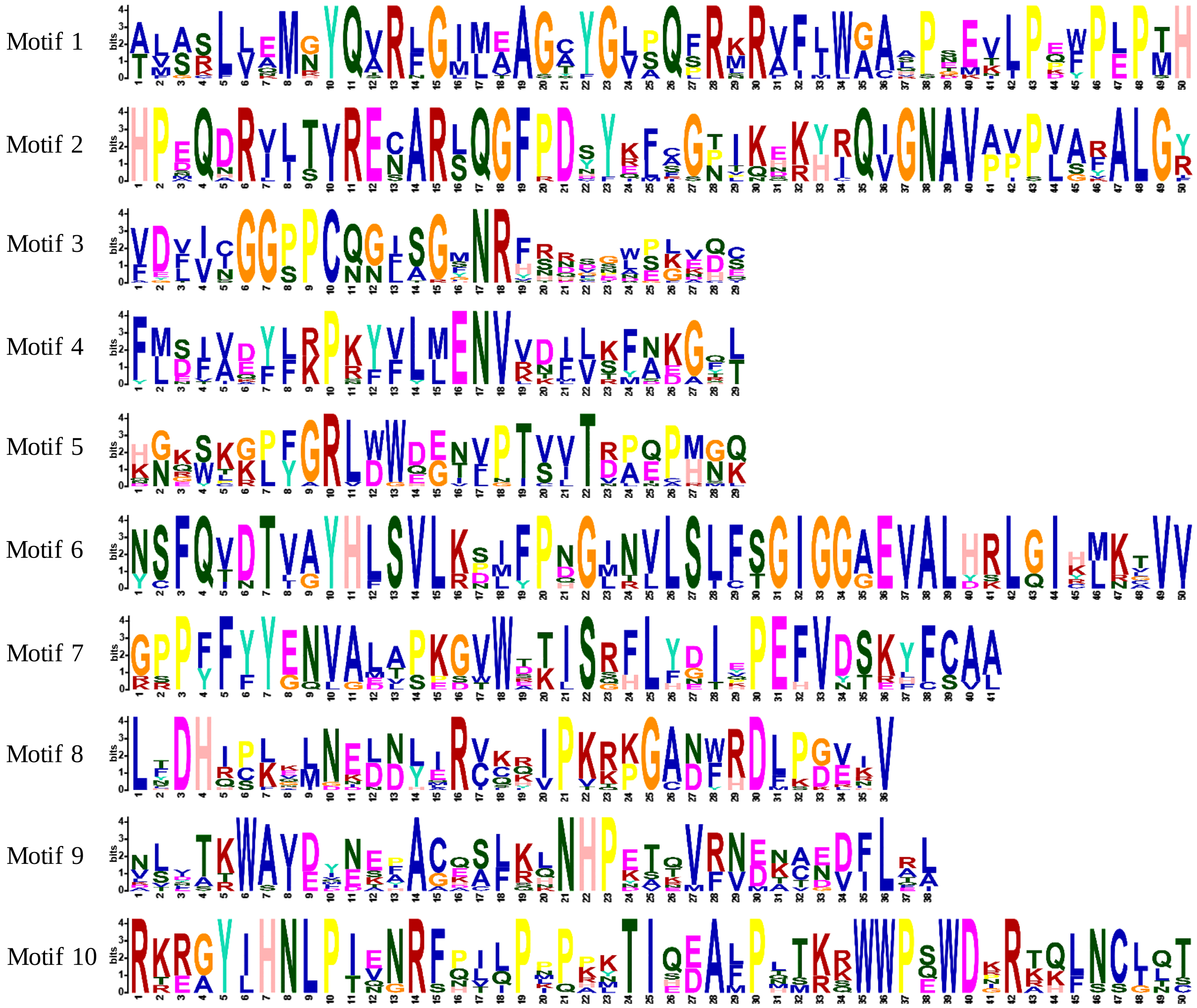

Supplemental Figure S7. Distribution of conserved motifs in DoC5-MTase based on the results of MEME analysis.
