# Supplementary material for: Genome-wide identification and analysis of DNA methyltransferase and demethylase gene families in Dendrobium officinale reveal their potential functions in polysaccharide accumulation
Source: BMC Plant Biol. 2021 Jan 6;21:21. doi: 10.1186/s12870-020-02811-8 (PMC7789594; doi:10.1186/s12870-020-02811-8)
Supplement: Supplementary file 15 — Additional file 15: Figure S9. Number of cis-elements in the promoter region of DoC5-MTase and DodMTase genes [file 12870_2020_2811_MOESM15_ESM.pdf]

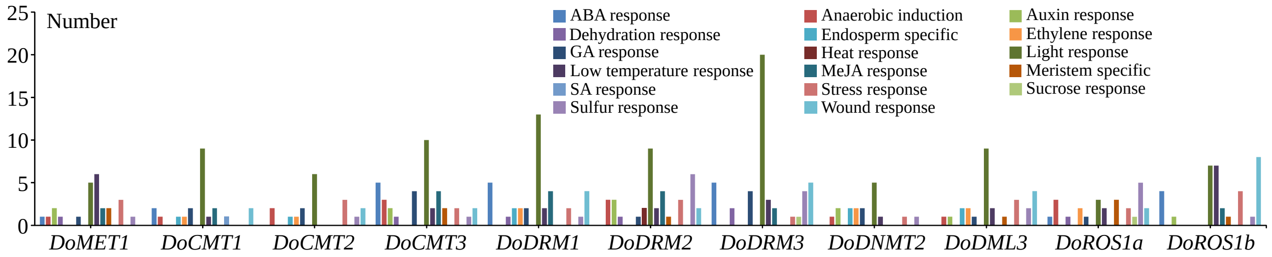

Supplemental Figure S9. Number of *cis*-elements in the promoter region of *DoC5-MTase* and *DodMTase* genes.
