# Supplementary material for: Genome-wide identification and analysis of DNA methyltransferase and demethylase gene families in Dendrobium officinale reveal their potential functions in polysaccharide accumulation
Source: BMC Plant Biol. 2021 Jan 6;21:21. doi: 10.1186/s12870-020-02811-8 (PMC7789594; doi:10.1186/s12870-020-02811-8)
Supplement: Supplementary file 16 — Additional file 16: Figure S10. The experimental scheme of D. officinale seedlings under sustained drought treatment and irrigation. Green arrows at 15:30 indicate the irrigation times, and magenta arrows indicate the experimental sampling times. White bars indicate light periods, and black bars indicate dark periods [file 12870_2020_2811_MOESM16_ESM.pdf]

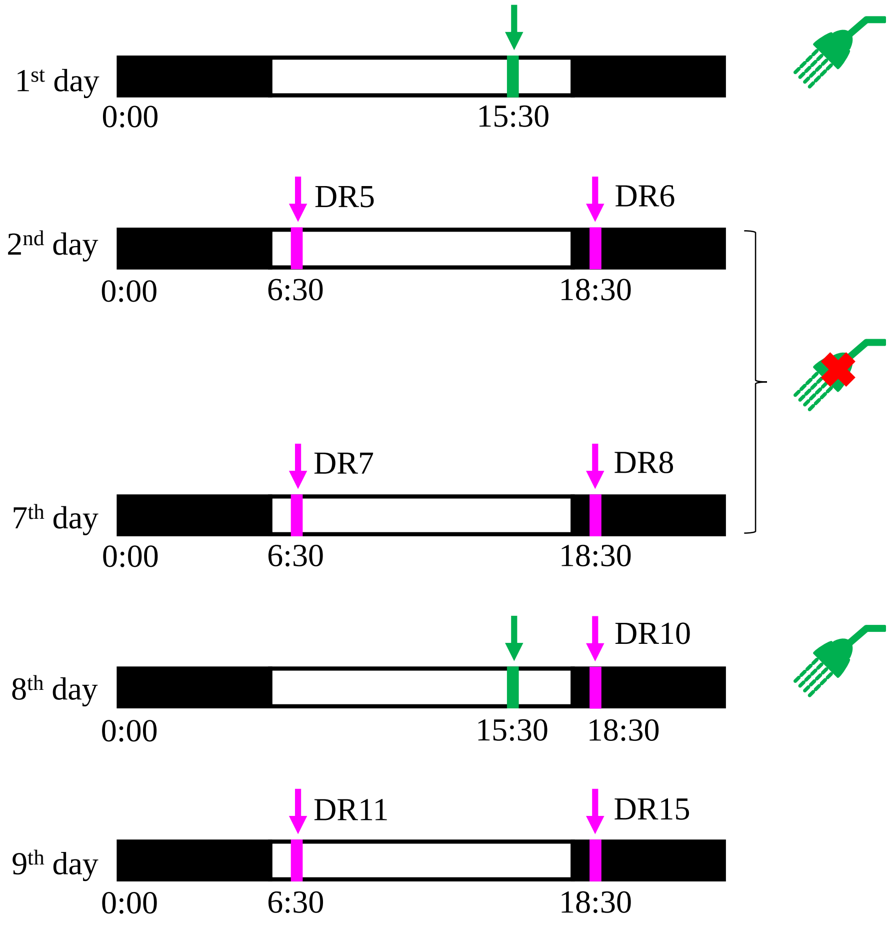

Supplemental Figure S10. The experimental scheme of *D. officinale* seedlings under sustained drought treatment and irrigation. Green arrows at 15:30 indicate the irrigation times, and magenta arrows indicate the experimental sampling times. White bars indicate light periods, and black bars indicate dark periods.
