# Supplementary material for: Genome-wide identification and analysis of DNA methyltransferase and demethylase gene families in Dendrobium officinale reveal their potential functions in polysaccharide accumulation
Source: BMC Plant Biol. 2021 Jan 6;21:21. doi: 10.1186/s12870-020-02811-8 (PMC7789594; doi:10.1186/s12870-020-02811-8)
Supplement: Supplementary file 17 — Additional file 17: Figure S11. WSP content in juvenile D. officinale stems at three different developmental stages (protocorm-like bodies, shoots and plantlets). (A-C) Three developmental stages (protocorm-like bodies, shoots and plantlets, namely S1, S2 and S3, respectively), which correspond to 2, 4 and 10 months after germination, respectively. (D) WSP content in protocorm-like bodies, shoots and plantlets of juvenile D. officinale stems. Error bars indicate the mean ± SD (standard deviation) of three individual experiments, and were performed in triplicate. Different letters above bars indicate a significant difference among different stages at p < 0.05 based on Duncan’s multiple range test. DW, dry weight. WSP, water-soluble polysaccharide [file 12870_2020_2811_MOESM17_ESM.pdf]

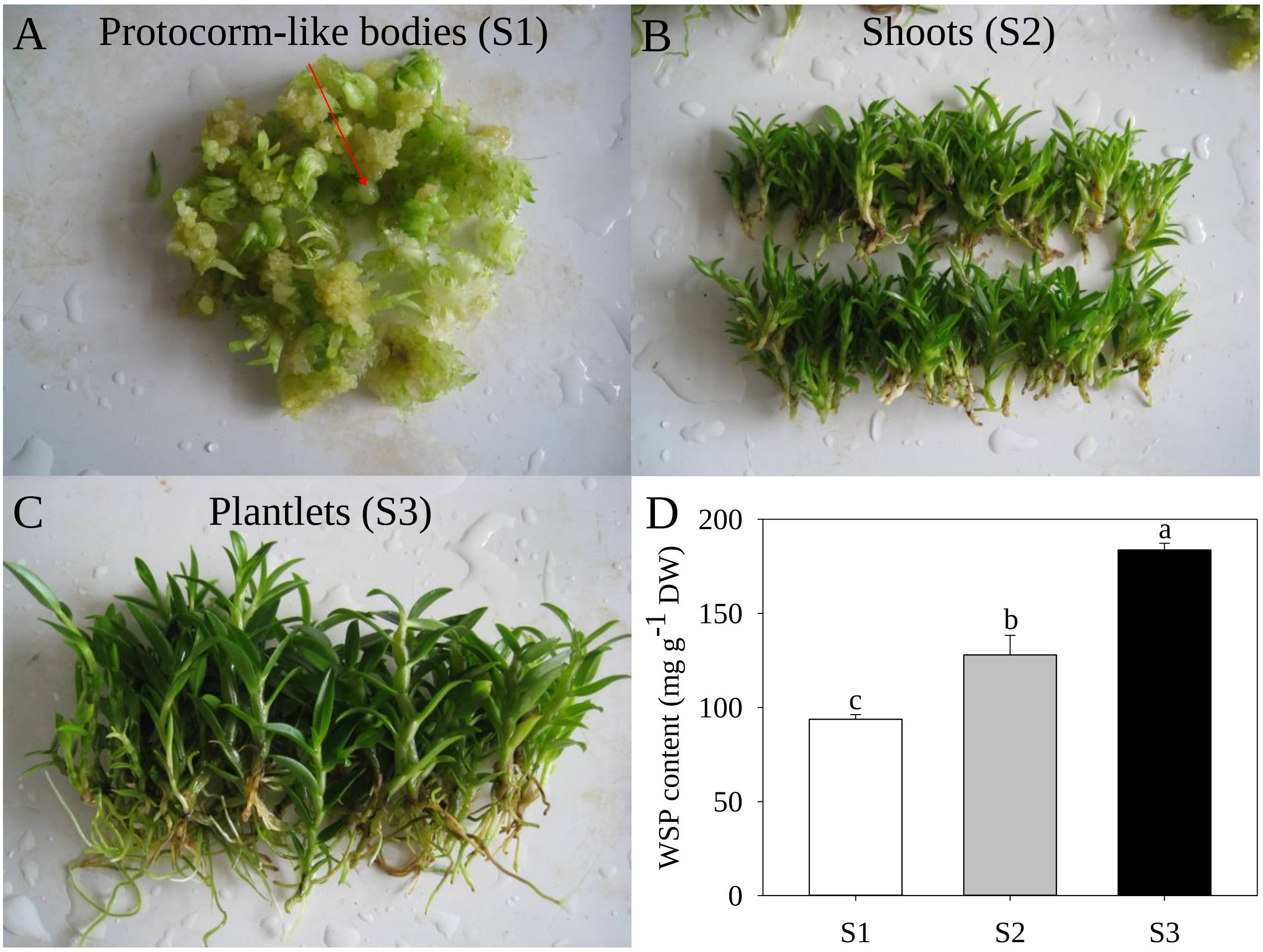

Supplemental Figure S11. WSP content in juvenile *D. officinale* stems at three different developmental stages (protocorm-like bodies, shoots and plantlets). Three developmental stages (protocorm-like bodies, shoots and plantlets, namely S1, S2 and S3, respectively), which correspond to 2, 4 and 10 months after germination, respectively. (D) WSP content in protocorm-like bodies, shoots and plantlets of juvenile *D. officinale* stems. Error bars indicate the mean ± SD (standard deviation) of three individual experiments, and were performed in triplicate. Different letters above bars indicate a significant difference among different stages at $p < 0.05$ based on Duncan's multiple range test. DW, dry weight. WSP, water-soluble polysaccharide.
